# Watching others in a positive state does not induce optimism bias in common marmosets (*Callithrix jacchus*), but leads to behaviour indicative of competition

**DOI:** 10.1007/s10071-021-01497-1

**Published:** 2021-03-16

**Authors:** J. E. C. Adriaense, V. Šlipogor, S. Hintze, L. Marshall, C. Lamm, T. Bugnyar

**Affiliations:** 1 Evolutionary Cognition Group, Department of Anthropology, University of Zürich, Zürich, Switzerland; 2 Department of Behavioral and Cognitive Biology, Faculty of Life Sciences, University of Vienna, Vienna, Austria; 3 Social, Cognitive and Affective Neuroscience Unit, Department of Cognition, Emotion, and Methods in Psychology, Faculty of Psychology, University of Vienna, Vienna, Austria; 4 Department of Zoology, University of South Bohemia, Budweiss, Czech Republic; 5 Division of Livestock Sciences, Department of Sustainable Agricultural Systems, University of Natural Resources and Life Sciences (BOKU), Vienna, Austria; 6 Bristol Veterinary School, Langford House, University of Bristol, Bristol, UK

**Keywords:** Emotional contagion, Cognitive bias, Judgement bias test, Animal emotions, Social cognition

## Abstract

Emotional contagion is suggested to facilitate group life by enhancing synchronized responses to the environment. Cooperative breeders are an example of a social system that requires such intricate coordination between individuals. Therefore, we studied emotional contagion in common marmosets by means of a judgement bias test. Demonstrators were exposed to an emotion manipulation (i.e., positive, negative, control), and observers perceived only the demonstrator’s behaviour. We predicted that the positive or negative states of the demonstrator would induce matching states in the observer, indicating emotional contagion. All subjects’ emotional states were assessed through behaviour and cognition, the latter by means of a judgement bias test. Behavioural results showed a successful emotion manipulation of demonstrators, with manipulation-congruent expressions (i.e., positive calls in the positive condition, and negative calls and pilo-erect tail in the negative condition). Observers showed no manipulation-congruent expressions, but showed more scratching and arousal after the positive manipulation. Concerning the judgement bias test, we predicted that subjects in a positive state should increase their response to ambiguous cues (i.e., optimism bias), and subjects in a negative state should decrease their response (i.e., pessimism bias). This prediction was not supported as neither demonstrators nor observers showed such bias in either manipulation. Yet, demonstrators showed an increased response to the near-positive cue, and additional analyses showed unexpected responses to the reference cues, as well as a researcher identity effect. We discuss all results combined, including recently raised validation concerns of the judgement bias test, and inherent challenges to empirically studying emotional contagion.

**Supplementary Information:**

The online version contains supplementary material available at 10.1007/s10071-021-01497-1.

## Introduction

Affective (emotional) mechanisms are assumed to underly many primate social behaviours (Schaffner and Aureli 2002; de Waal 2011), such as allogrooming (Russell and Phelps 2013; Schino et al. 2016), offspring care (Preston and de Waal 2002), and affiliative bonding (Aureli and Schino 2004), as well as self-directed behaviours elicited by social interactions (e.g., self-scratching, Troisi et al. 1991). Social processes, such as emotionally mediated reciprocity (Aureli and Schaffner 2002; Schino and Aureli 2009), fairness (Yamamoto 2012), and cooperation (Massen et al. 2019), have also been suggested to be underpinned by affect-based mechanisms. Emotional contagion is suggested as one of the more fundamental affective mechanisms of empathy (de Waal 2008), and of other empathy-related and social behaviour such as social learning and affect-based helping (see Adriaense et al. 2020, for review). It is defined as an emotional state-matching between individuals (Hatfield 1994; Preston and de Waal 2002), and as such, it does not imply a cognitive representation of, or concern for, the other’s emotional state (e.g., Adriaense et al. 2019b). Functionally, it is proposed to facilitate group life through fast, emotion-based responses which enhance information transmission (Nakahashi and Ohtsuki 2018), improve social interactions and affiliative bonding, and increase defence against predation (Preston and de Waal 2002; Decety 2015; Isern-Mas and Gomila 2019). For this reason, the study of emotional contagion is of particular interest in group-living (e.g., in pigs, Reimert et al. 2017) and pair-bonding species (e.g., in prairie voles, Burkett et al. 2016; in common ravens, Adriaense et al. 2019a).

Here, we argue that emotional contagion is also one of the potential affective mechanisms in cooperative breeding species (Massen et al. 2019). In particular, this reproductive system, where non-parents help in taking care of offspring (e.g., in humans, Kramer 2010; in primates, Martin et al. 2021), requires efficient communication, intricate spatial and temporal coordination between group members, as well as an increased attention to others (Burkart et al. 2009). Common marmosets are cooperative breeders of the callitrichid family, and in their social allo-parenting system the dominant breeding pair lives together with their offspring and non-breeding adult helpers (Digby and Barreto 1993; Erb and Porter 2017; Schiel and Souto 2017). Aside from this cooperative parental care, marmosets also show cooperative territorial defence (Lazaro-Perea 2001), and, as mentioned, these cooperative behaviours require efficient coordination and group cohesion (Burkart and van Schaik 2010; Massen et al. 2016). In particular, it is suggested that social skills are important (Burkart et al. 2009; Burkart and van Schaik 2009), and common marmosets demonstrate high degrees of prosociality (Burkart et al. 2014; Martin et al. 2021), including unsolicited prosociality toward non-reciprocating and unrelated individuals (Burkart et al. 2007). Therefore, we suggest that in common marmosets, emotional contagion is essential for cooperation.

To empirically test emotional contagion and establish an appropriate interpretation, it is important to assess the emotional states of both the sender and the receiver, and verify whether their states match (Adriaense et al. 2020, for review of emotional contagion). An emotional state is suggested to orient on two dimensions, namely arousal (i.e., low or high intensity) and valence (i.e., positivity or negativity) (Russel 1980; Mendl et al. 2010), and thus, matching emotional states ought to reflect similarity on both dimensions. This is an important notion, as matching arousal in two individuals does not necessarily imply matching valence, and vice versa (Briefer 2018). For instance, increased heart rate is indicative of high arousal but not necessarily of positive (e.g., excitement) or negative (e.g., fear) valence (Edgar et al. 2012). In that vein, matching (synchronized) behaviours or physiological expressions do not unequivocally imply matching emotional states (see Massen and Gallup 2017, for review of yawn contagion; see Isern-Mas and Gomila 2019, for review of the mimicry mechanism; see Adriaense et al. 2020, for review of play contagion). Moreover, measuring valence is considered more difficult than measuring arousal, and research shows that previously assumed measures of valence in fact measure arousal (Paul et al. 2005; MacDougall-Shackleton et al. 2019). Considering this, researchers in the animal domain have to be additionally cautious, as a major obstacle in this field is the absence of (human) language to provide self-report on the subjective emotional experience (Paul et al. 2005). In conclusion, empirically observing behavioural or physiological synchronization in animals cannot be taken as definite evidence for emotional contagion. This does not imply that behavioural or physiological observations do not greatly contribute to our understanding of animal emotions (Paul et al. 2005), or that mimicry does not play an important role in emotional contagion (Lakin et al. 2003) or in social relations (McIntosh 2006), but rather that interpretations of emotional contagion should not depend on observing synchrony alone, and that additional objective indicators are needed.

Emotional contagion in common marmosets has not been directly investigated yet, though its presence has been suggested (Finkenwirth et al. 2015). Furthermore, synchronized responses have experimentally been observed in coordinated behaviour during joint action tasks (Miss and Burkart 2018), in behavioural contagion such as contagious scent-marking and gnawing (after visual demonstration of a conspecific, Massen et al. 2016) and contagious affiliative expressions (after auditory demonstration, Watson et al. 2010), as well as in synchronized oxytocin fluctuations over time in strongly bonded dyads (Finkenwirth et al. 2015). Inferring emotional contagion should ideally be based on the assessment of multiple indicators which allow interpretation of both dimensions of an emotion (i.e., arousal and valence) (Mendl et al. 2010). In that regard, emotions are considered adaptive, multi-componential, and global responses to the environment, causing coordinated changes in behaviour, physiology (incl. neurology and endocrinology), cognition, and feelings (Paul et al. 2005; Anderson and Adolphs 2014). This functionalist approach, in which emotions are considered central states (Adolphs and Andler 2018), allows animal emotion research to focus on the objectively measurable components, and sets aside the conscious feeling component, allowing for systematic, comparative research (Anderson and Adolphs 2014). Usually, behaviour and physiology are more often studied as potential indicators of animal emotions, than the cognitive component. Yet, recent developments of the so-called cognitive bias paradigm offer a promising method to not only incorporate the cognitive component, but also provide a means to measure valence (Harding et al. 2004; Mendl et al. 2009). The paradigm is based on the emotion-cognition interaction premise (Pessoa 2013), which finds support in neuroscience (Clore 2018) as well as in human psychology and psychiatry. This support demonstrates that cognitive processing may alter emotions (e.g., by means of appraisal) and that emotions may induce cognitive changes, referred to as cognitive biases (i.e., *bias* here implies an impact or influence, rather than error). Positive or negative emotional states bias cognitive processing in a congruent manner, so that memory, attention, or decision-making will either be more positively, or negatively, biased, respectively (i.e., negative states induce, e.g., negative decision making, Eysenck et al. 1991; negative future anticipation, MacLeod and Byrne 1996; negative attention, Mathews and MacLeod 1994; and vice versa for positive states, e.g., Eysenck et al. 1991; Nygren et al. 1996). Similarly, by analysing an animals’ cognitive performance under specific conditions, we may find cognitive biases in their responses (Paul et al. 2005; Mendl et al. 2009), which may serve as a proxy to assess the subject’s emotional valence (Neville et al. 2020; Lagisz et al. 2020). The bias hypothesis predicts that animals in a positive state should show a positive or ‘optimism’ bias, and animals in a negative state should show a negative or ‘pessimism’ bias (note that this not implies a subjective experience of optimism or pessimism, Lagisz et al. 2020).

The judgement bias test (JBT) is one of the most frequently used cognitive bias designs, which measures biases in decision making under ambiguity. Typically, in this paradigm, animals are trained to associate one cue with a positive reward (i.e., the positive cue) and another cue with no reward or a punishment (i.e., negative cue). After successful training, animals are then presented with (an) untrained, ambiguous cue(s). Here, the animal’s response to the ambiguous cue(s) is measured and whether this response *biases* more toward the response given to the positive cue (e.g., by faster response time or more responses) or to the negative cue (e.g., by slower reaction time or fewer responses). The JBT has been applied across a wide range of mammalian, avian, and invertebrate species (see for reviews: Mendl et al. 2009; Bethell 2015; Roelofs et al. 2016; Neville et al. 2020; Lagisz et al. 2020). The majority of these studies focused on a focal animal, and assessed whether a presumed change in affect due to, for instance, husbandry procedures (e.g., enriched environment, Douglas et al. 2012; social housing, Lalot et al. 2017), corresponds with the predicted bias in a JBT. The interaction between experimentally induced shifts in affect and related biases in cognitive performance has also been successfully studied in a number of monkey species. Research in rhesus macaques found effects of husbandry procedures, providing evidence for associations between environmental enrichment and optimism bias, and between a veterinary visit and pessimism bias (Bethell et al. 2012). Moreover, in capuchin monkeys, stereotypical behaviour such as head twirls (but not pacing) correlated with pessimism bias, together with higher corticoids levels (Pomerantz et al. 2012). Monkeys who show overall higher rates of scratching also show less optimism bias (Schino et al. 2016), and individuals that generally receive more grooming and rank as alpha male, show more optimism bias (Schino et al. 2016). In common marmosets, no previous work has studied the relation between experimentally induced states and cognitive bias, though two studies used a judgement bias paradigm to assess effects of rearing (hand-reared monkeys showed no bias when compared to family reared ones, Ash and Buchanan-Smith 2016) and effects of handedness (left-handed monkeys showed pessimism bias and received more group aggression, Gordon and Rogers 2015) (see also Perdue 2017, for bias results with no experimental manipulation in rhesus and capuchin monkeys). In apes, the use of a JBT has so far been successful in one study (in terms of reaching training criterion). JBT was investigated in three chimpanzees, and whether general tendencies to expect reward or not, could potentially serve as a source to assess poor welfare when overt expression is missing (Bateson and Nettle 2015). The study did not use an experimental manipulation, but results showed individual variance remaining stable over 2 weeks. The bias methodology is not always easily transferred between species, and repeated research in gorillas showed that subjects either were not able to pass the required discrimination learning (note the small sample of three subjects) or exhibited individual differences putting the findings into question (McGuire et al. 2017; McGuire and Vonk 2018).

To our knowledge, only two studies so far have used a JBT to assess emotional states stemming from emotional contagion, namely in rats (Saito et al. 2016) and in common ravens (Adriaense et al. 2019a). Saito and colleagues (2016) used a playback experiment with a go/go JBT. First, rats were trained to press lever A upon hearing one sound (i.e., positive cue) to receive a reward, and to press lever B upon hearing the other sound (i.e., negative cue) to avoid hearing a punishing white noise. After training, rats underwent a 20-min playback of either positive or negative vocalisations of conspecifics, serving as emotional contagion manipulation, followed by a JBT. Here, the two trained sounds were presented with in addition three ambiguous sounds (i.e., near-positive, middle, near-negative), and with each sound the rats were given the option to press either lever A or B. Results show that after hearing positive calls, and presented with an ambiguous middle cue, rats pressed the rewarding lever more (compared to the control and negative calls). Yet, in contrast to the predictions, after hearing, the negative calls rats did not press the avoidance lever more than in the control condition. When presented with the near-negative cue, rats pressed the avoidance lever more in the negative condition (compared to the control and positive calls), but after hearing the positive calls, they did not press the reward lever more (compared to the control condition). Importantly, as the middle-cue responses to the control calls were similar to the negative calls, it remains difficult to infer whether the found effects were due to the treatment, due to another unknown variable, or were enhanced due to a confounded control condition. Here, adding a pre- and post-manipulation test would allow to hone in on changes specifically due to the treatment. The second study (Adriaense et al. 2019a, b) used a demonstrator and observer set-up with a spatial go/no-go JBT. First, ravens learned that pecking a box on one side lead to receiving a reward (i.e., positive cue), and that pecking a box on the opposite side lead to no reward (i.e., negative cue). After training, ravens participated in an emotional contagion experiment with a demonstrator being exposed to a two-minute positive or negative emotion manipulation, and an observer watching the demonstrator. This was followed with a JBT where a box was presented on the two trained sides with in addition a new ambiguous location (i.e., in the middle). As expected, results of the negative condition showed that after watching the demonstrator, observers pecked the box on the ambiguous location less (compared to both the positive and baseline pre-test). This indicates that ravens perceived the ambiguous cue as less rewarding when the other raven was in a negative manipulation, though, no change in pecking was found in the positive manipulation (when compared to its pre-test).

In our study, we investigated emotional contagion in common marmosets by means of a demonstrator and observer design with a go/no-go JBT (similar to Adriaense et al. 2019a). Our research thus contributes to the further validation of the bias paradigm, and the scientific investigation of affective mechanisms underlying cooperative breeding systems. To follow the recommended multi-component approach in emotion research, and provide an additional validity check of the JBT results, we also measured both subjects’ behaviour and vocalisations throughout the entire experiment. This design allowed us to assess the emotion manipulation effect (i.e., by means of the demonstrator’s behaviour and judgement bias data), as well as emotional contagion (i.e., by means of the observer’s behaviour and judgement bias data).

## Methods

### Sample

In total, 8 common marmosets (four females, four males) participated in this study. An additional five marmosets participated in the training prior to testing, but they did not reach criterion within the designated study timeframe. All animals were born in captivity and housed at the Department of Behavioral and Cognitive Biology, UZA 1, University of Vienna, Austria (see SI Table S1 for subject details; see SI for housing details).

### Procedure

**Fig. 1:**
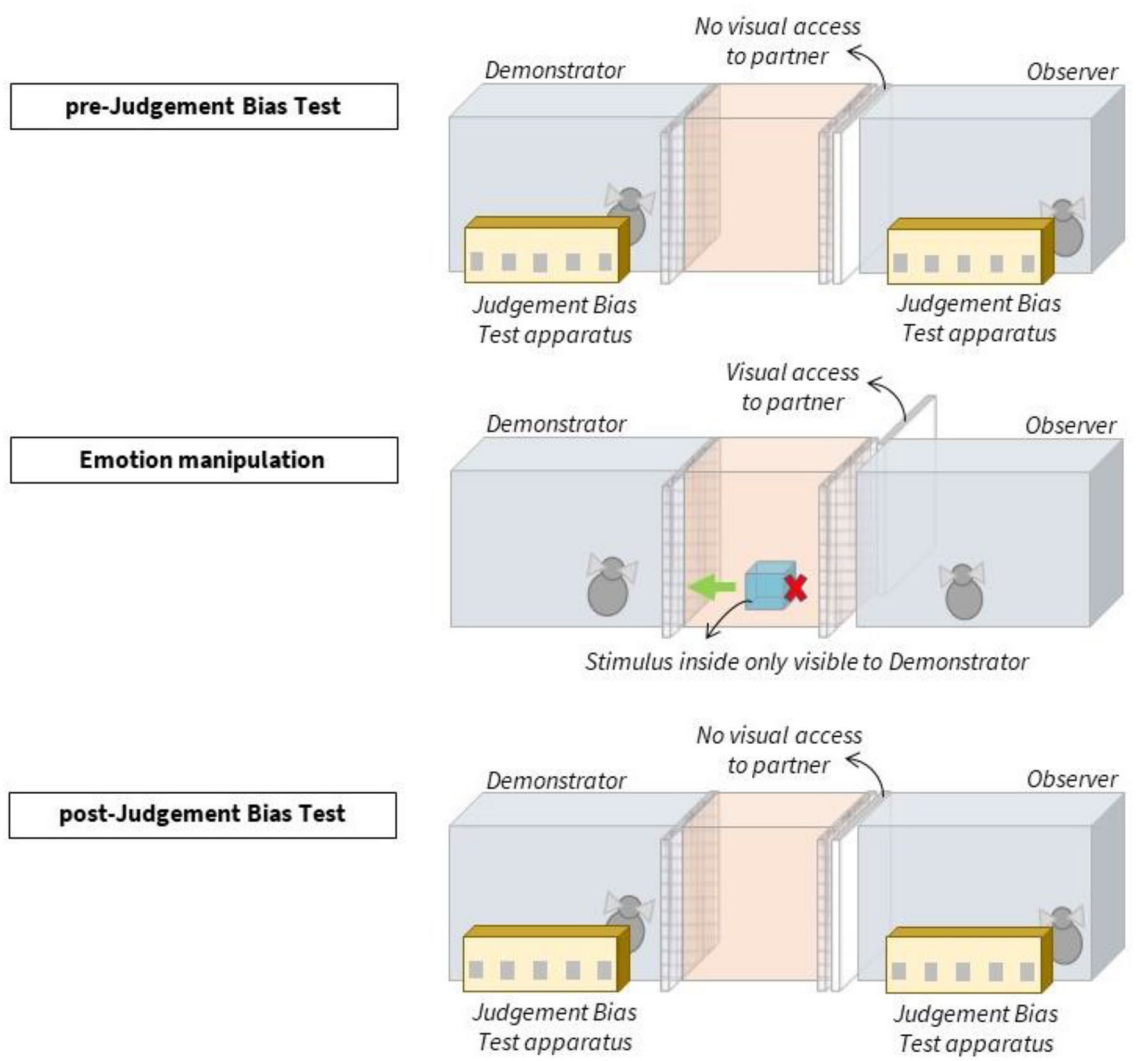
*Experimental design*. Subjects participated in dyads. During the pre- and post-JBT, a moveable door was closed (to not distract the subjects from seeing their partner), and each subject was tested on a JBT at the same time, and in separate compartments. During emotion manipulation, the demonstrator had visual access to the contents of a box placed in the middle compartment. The observer could not see the contents, as the box opening was directed toward the demonstrator, but was able to observe the demonstrator’s behaviour (see also SI Fig. S1)

One test session consisted chronologically of a pre-JBT, an emotion manipulation, and a post-JBT (see Fig. 1). Subjects participated in dyads with one demonstrator (*n* = 8) and one observer (*n* = 7, three females, four males). During both the pre- and post-JBT, the pair was tested at the same time and each animal underwent the test by one researcher (i.e., JA and VŠ) (see Fig. 1; SI Figs. S1, S2, S3). The pre-JBT served as baseline measurement to compare the post-JBT results to, and thus, to provide both within- and between-condition comparisons (average time per JBT: 10 min). During emotion manipulation, the demonstrator was exposed to either a positive or negative stimulus, or a control (presentation time: 2 min), while the observer was exposed to the demonstrator’s behaviour only and could not see the stimuli used for the manipulation. After the post-JBT the subjects were free to join their social groups again (average time per session: 22 min) (see SI for details).

**Total test sessions**. Each subject (excl. Aurora, see SI for details) participated in total in 12 test sessions; twice as demonstrator and twice as observer in all three conditions. These repeated measures (i.e., labelled ‘period 1′, ‘period 2′ in our variables; see SI) were introduced as to verify whether the emotion manipulation would have stronger or weaker effects over time. We only conducted period 2 testing once the subjects had completed all testing of period 1 (i.e., once as demonstrator in all three conditions and once as observer in all three conditions).

**Emotion manipulation**. After the pre-JBT, a researcher placed a foam box inside the middle compartment, with its one opening directed toward the demonstrator (see Fig. 1). In the positive condition, the box contained a bowl of banana pieces (i.e., preferable food item, to induce a positive state), in the negative condition an artificial large rubber spider (i.e., threatening item, to induce a negative state), and in the control condition the box remained empty (see SI Fig. S4).

**Judgement bias paradigm.** A judgement bias paradigm consists of a discrimination training and then a judgement bias test. Due to the nature of this paradigm and the use of ambiguous cues, various elements of the methodology are important to consider when designing a bias test. When these elements are not appropriately incorporated, they may inflate findings of either pessimism or optimism biases, or the test may measure other variables (e.g., response to novelty) rather than response to ambiguity. Below, we describe how we incorporated these requirements.

**Judgement bias training: hybrid go/no-go design**. A key element of a JBT is the choice of either a go/no-go or a go/go design (Mendl et al. 2009; Brilot et al. 2010; Roelofs et al. 2016). The former is commonly used and refers to the animal either actively approaching a cue or not at all. Yet, a known limitation is the requirement for response suppression as subjects need to actively inhibit their behaviour when being exposed to the presented cue, which is known to be difficult (Mendl et al. 2009). As a result, such inhibition issues may during testing either lead to (seemingly) optimistic or pessimistic responses, which in reality may be either failures to inhibit, or to react, respectively. The go/go design obligates the subjects to make active choices and, thus, the design does not require inhibition. Still, this design is more cognitively demanding and its training often needs more time to reach criterion (Roelofs et al. 2016; Lagisz et al. 2020). Therefore, we used a hybrid go/no-go design which includes a third choice serving as opt-out alternative (Hintze et al. 2018). Concretely, with this design, subjects have the choice to either approach the presented cue or not, and when they do not, they have the choice to opt-out and start the next trial, or do nothing and wait out the intra-trial time. This opt-out alternative (i.e., “trial initiator”) also limits the requirement for inhibition and puts the animal in control of the trial progress. Due to this addition, the training prior to the experiment consisted of two stages, namely apparatus training and discrimination training.

**Judgement bias training: apparatus training.** We used a spatial JBT (similar to Hintze et al. 2018) with five horizontally placed doors as cues. The doors could be opened and closed by a researcher, and upon opening the door hit an object creating a sound. We implemented this auditory cue in addition to the visual cue (i.e., actual opening of door) to increase the saliency of an open door, and thus, to ensure that subjects were attentive to the cues during testing (Hintze et al. 2018). During apparatus training, animals were trained to touch the trial initiator as necessary requirement before any of the JBT cue doors would be opened. Concretely, each trial began when subjects touched the trial initiator, which then went out of view for 3 s, and, simultaneously, one of the two reference doors of the apparatus (i.e., the positive, “P”, and negative, “N”, cue) would be opened. The subjects had three options within this trial: approach the open door within 10 s (coded as go response), do not approach the open door within 10 s (coded as no-go response), or touch the trial initiator again after it was unavailable for 3 s (coded as no-go and active choice) (see SI Table S3 for ethogram). With this third choice, a new trial began immediately, in which the previous door closed and a new one opened. If within this trial, the subjects did not approach the open door and also did not touch the initiator, the open door would be closed after 10 s and a new trial would only start when the subject touched the initiator (see SI for training details; SI Table S2 for training schedule; and Figs. S2, S3 for details).

**Judgement bias training: discrimination training.** After apparatus training, subjects learned that an open door on one side would provide a food reward upon approach (i.e., P cue), and an open door on the other side (counterbalanced) would never provide a reward upon approach (i.e., N cue). The criterion to pass discrimination training was set at 80% go response for P cues (i.e., approach the open P door within 10 s) and 80% no-go response for N cues (i.e., do not approach the open N door within 10 s, or initiate a new trial within 10 s), calculated per day, over 3 consecutive days (See SI Table S2).

**Judgement bias test.** After learning the reward values of the two reference cues, resulting in an overall approach of P and avoidance of N, subjects participated in the experiment with a pre- and post-JBT. During both JBTs, subjects were presented with the reference cues P and N, with addition of three new ambiguous cues. We predicted that demonstrators undergoing a positive manipulation should in the post-JBT respond to the ambiguous cues in a similar way as to the trained positive cues (i.e., more go responses, as an indication of optimism bias). Correspondingly, after the negative manipulation, subjects should in the post-JBT respond more similarly as to the negative cues (i.e., fewer go responses, pessimism bias). Through processes related to emotional contagion, we expected observers to show the same pattern. Importantly, ambiguous cues should be equally related to the reference cues (Roelofs et al. 2016). Therefore, we chose a gradual, horizontally oriented design going from the reference cue P to the near-positive cue (NP), a middle cue (M), near-negative cue (NN), and the reference cue N (see SI Fig. S2). Furthermore, multiple measurements over time may lead to learning the true reward value of the ambiguous cues. This causes a loss of ambiguity, and eventually may result in inflating findings of optimism or pessimism bias. We incorporated four solutions to prevent and account for a potential learning effect. First, NP, M, and NN were only presented once per JBT, as a lower amount decreases the possibility of learning (Roelofs et al. 2016). Second, we added a control condition and a pre-JBT to provide baseline results and to assess the response to ambiguity in the absence of experimental manipulation, in which no response change is expected. Third, NP, M, and NN trials were rewarded when approached (same as in Hintze et al. 2018). Detected learning effects often concern inflated findings of pessimism biases, potentially due to the saliency of unrewarded ambiguous cues. Hence, using a reward schedule may be less salient, and thus, may lead to reduced learning. And fourth, we statistically accounted for a chronological order effect of the ambiguous cues (see SI for further details; and SI Table S4 and S5).

**Behavioural responses.** JBT results should be further validated with other variables which are assumed to assess affect (e.g., Rygula et al. 2012). Therefore, we quantified behaviour shown during the entire experiment. During the positive emotion manipulation, we predicted to observe positive state-related behaviour. Specifically, we focused on vocalisations related to food anticipation (from here on labelled ‘positive calls’), such as “chirp” and “food-beg" calls (e.g., Epple 1968; Watson et al. 2010). We also expected subjects to position themselves in front of the stimulus (labelled ‘position’) more than in the negative condition, and this for both demonstrators and observers. The positive stimulus was visually oriented toward the demonstrator’s compartment, and thus, we expected them to try to position themselves as close as possible to this stimulus. Due to the increased attention of the demonstrator to this location, we predicted that observers would also position themselves in front of the box (though from the other side without visual access). During the negative emotion manipulation, we predicted negative state-related behaviour. We focused on predator or alarm related calls (‘negative calls’), such as “tsik”, “tsik-egg”, “cough”, and “seep” calls, which are described as mobbing or alarm calls in reaction to threat or predation (Bezerra and Souto 2008). We also expected subjects to position more elsewhere in the cage, and exhibit more pilo-erected tail (‘pilo-erect tail’) and scratching (‘scratching’). In marmosets, pilo-erected tail is often a behavioural indicator of elevated arousal (see Brügger et al. 2021, for association with nasal temperature decrease), with some reporting correlations with negative events (Ermatinger et al. 2019), and scratching is frequently a measure of (negative) stress (e.g., Bassett et al. 2003). Through emotional contagion, we predicted to find similar patterns of these calls and behaviours in the observers in the respective conditions. Furthermore, we expected that some of these behaviours would persist during the post-JBTs, although they would likely be less present than during the manipulation, as animals were assumed to be more occupied with responding to the test. For overall additional exploratory purposes, we looked at other behaviours shown during the manipulation and the JBTs, such as scent-marking, gnawing, contact calls (i.e., “phee”, “shrill”, and “whirr”), “egg” calls, defecating, urinating, and self-grooming (see SI Table S3 for ethogram).

**Recording.** The entire study was video recorded and afterwards the files were re-named to ensure blinding of the data. An independent researcher (LM), who was unaware of the research questions at the time, used the re-named files to code all go/no-go responses and behaviour during JBTs. JA used the re-named files to code the behaviour during the emotion manipulation, and VŠ recoded 15% of all behaviour and 15% of go/no-go responses. Interobserver reliability was assessed using a fixed-effects, single-observer intra-class correlation coefficient (ICC). Reliability was found to be high across all parameters: go/no-go responses during JBTs, ICC(3,1) = 0.99, behaviour during JBTs, ICC(3,1) = 0.98, and behaviour during manipulation, ICC(3,1) = 0.90. All videos were coded using Solomon Coder software (Péter 2017).

**Statistical analyses**. All analyses were done in R 3.6.2 statistical environment (R Core Team 2019) and we used generalized linear mixed models (GLMM’s), using the ‘lme4′ package (Bates et al. 2015). GLMMs account for repeated measures within subjects, enhance statistical power, and avoid artificial reduction of the variability in the dataset (Gygax 2014). For all models, we used a likelihood ratio test and the Akaike’s information criterion (AIC) as model selection procedure to identify the best model explaining variation in the dependent variables, go response, and behaviour. The likelihood ratio test verifies whether two models significantly differ from each other, and when no significant difference is shown, the least complex model is chosen. If a significant difference exists, the AIC values of the two models are compared, and when the difference is less than 2, both models are considered sufficient and the least complex model is chosen, and when the difference is 2 or higher than 2, the model with the lowest AIC is considered better (Symonds and Moussalli 2011). Based on the variability observed between individuals for overall go responses in the JBT (see SI Fig. S5), we included *subject* as random variable in all our subsequent analyses, as well as *date*.

For our main research question (i.e., optimism or pessimism bias) and, thus, to assess differences in go responses between (i.e., between positive, negative, control) and within (i.e., between pre- and post-JBT) each condition, the model was specified with a binomial distribution using logit transformations. We included *test* nested within *cue* nested within *condition* as this specific interaction was expected to show differences in the go response. Due to our repeated-measures study design, we expected different results in the repeated measures, so we included *period* (i.e., period 1, period 2) as predictor. For theoretical reasons regarding emotional contagion, we included *role* (i.e., demonstrator, observer), and for exploratory purposes, we included *researcher* (i.e., VŠ, JA) and *time* (i.e., testing in the AM, testing in the PM) as predictors, as well. A likelihood ratio test and AIC comparing this full model to a reduced model (i.e., without the main or interaction effects), supported a model with *researcher*, *role*, and *period* each as main effect, and *condition/test/cue* as interaction.

Each basic behaviour model included *condition* (positive, negative, control) as predictor, and during model comparison, this was compared to a full model with either predicted or exploratory variables (see SI for details). To verify differences in subjects’ behaviour during emotion manipulation, the model was specified with a Poisson distribution using log-link transformations for behaviour counts (i.e., all vocalisations, scratching, scent-marking, gnawing), and a binomial distribution with logit transformation for pilo-erected tail. We also coded position during emotion manipulation, for which we performed a logit transformation on the proportion of time spent in front. For behaviour during emotion manipulation, we compared mean behaviour counts between and within conditions. To assess differences in behaviour during JBTs, we included *condition/test* as interaction effect, and the model used a Poisson distribution for behaviour counts (i.e., scratching, scent-marking, gnawing), with comparisons made between and within conditions (see SI for final models for each behavioural parameter).

## Results

On average, marmosets reached criterion for discrimination training in 6 days and 128 trials (range days: 3–9; range trials: 60–180) (see SI Table S4 and S6).

### JBT discrimination training success

**Fig. 2:**
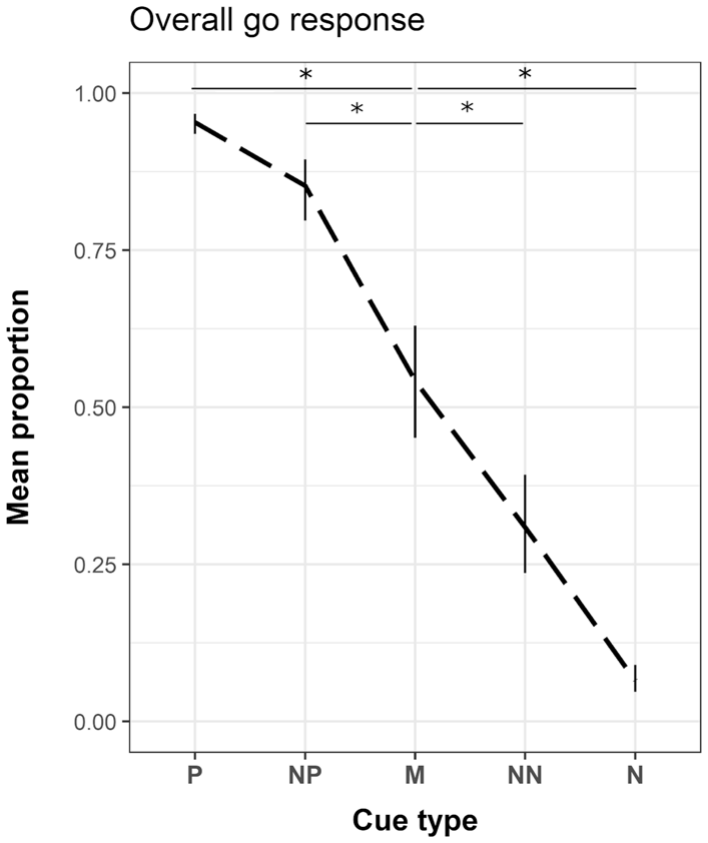
Mean predicted proportion (bars indicate SE) of go responses to the five cues of the JBT. Plot shows overall result of all subjects, across all conditions, confirming a successful discrimination training between the different reference cues and their reward values, and the required monotonically graded response. *P *positive, *NP *near-positive, *M *middle, *NN *near-negative, *N *negative. *P ≤ 0.001

We verified discrimination training success and the monotonically graded response pattern as a means of internal validity (Hintze et al. 2018). To this end, we ran a model with *cue* as independent variable (with *subject* and *date* as random variable) and found that subjects correctly exhibited a significantly higher proportion of go responses to the *P* cues (compared to M: β = 2.854, z = 12.602, *P* < 0.001), and a significantly lower proportion of go responses to the N cues (compared to M: β =  – 2.836, z =  – 12.893, *P* < 0.001). Subjects also responded to the intermediate cues as being gradually different from M with a significant higher proportion for NP (compared to M: β = 1.586, z = 5.885, *P* < 0.001), and a significant lower proportion for NN cues (compared to M: β =  – 0.973, z =  – 3.960, *P* < 0.001). This gradual decline in go responses from the P to the N cue shows the monotonic graded response curve ideally observed in JBT results (Gygax 2014) (see Fig. 2 for group-level results, and SI Fig. S5 for individual-level results), which confirms that the animals successfully learned to discriminate between the P and N cues, as well as that NP, M, and N were perceived as intermediate (see Fig. 2).

The use of the trial initiator showed a similar monotonic response pattern, resulting in a low proportion to opt out for the P cue (4%), followed by a gradual incline between the intermediate cues NP (10%), M (26%), and NN (42%), to a higher proportion opting out for the N cue (70%). This confirms the use of the trial initiator as active choice aside from a go response. On average during testing, the P cue responses had a 6.7% error rate (i.e., performing an incorrect no-go response) and the N cues 8.4% (i.e., performing an incorrect go response), which is in line with the other reports, and thus, confirms the success of our training (e.g., error rate in horses for *P*: 6.03%; for N: 13.19%; in mice for P: 2.40%; for N: 10.60%; in rats for *P*: 1.37%; for N: 12.62%, Hintze et al. 2018; error rate in calves for *P*: 1.44%; for N: 10.24%, Buckova et al. 2019).

### JBT learning effect of ambiguous cues

Then, we verified whether the go response to the ambiguous cues changed over time, potentially indicating a learning effect of its reward value. We added *order* as independent variable (with *subject* and *date* as random variables), and we found that over time there was no significant change in go responses to specifically the ambiguous cues (for NP; β =  – 0.026, z =  – 0.608, *P* = 0.543; for M; β =  – 0.017, z =  – 0.552, *P* = 0.581; for NN: β =  – 0.061, z =  – 1.728, *P* = 0.084). This confirms that subjects did not learn the reward value of either ambiguous cue, and that a potential optimism bias would not have been due to an order effect. For that reason, we did not add *order* as control variable in our subsequent analyses.

### Go responses during JBT

#### Responses to the ambiguous cues

**Fig. 3:**
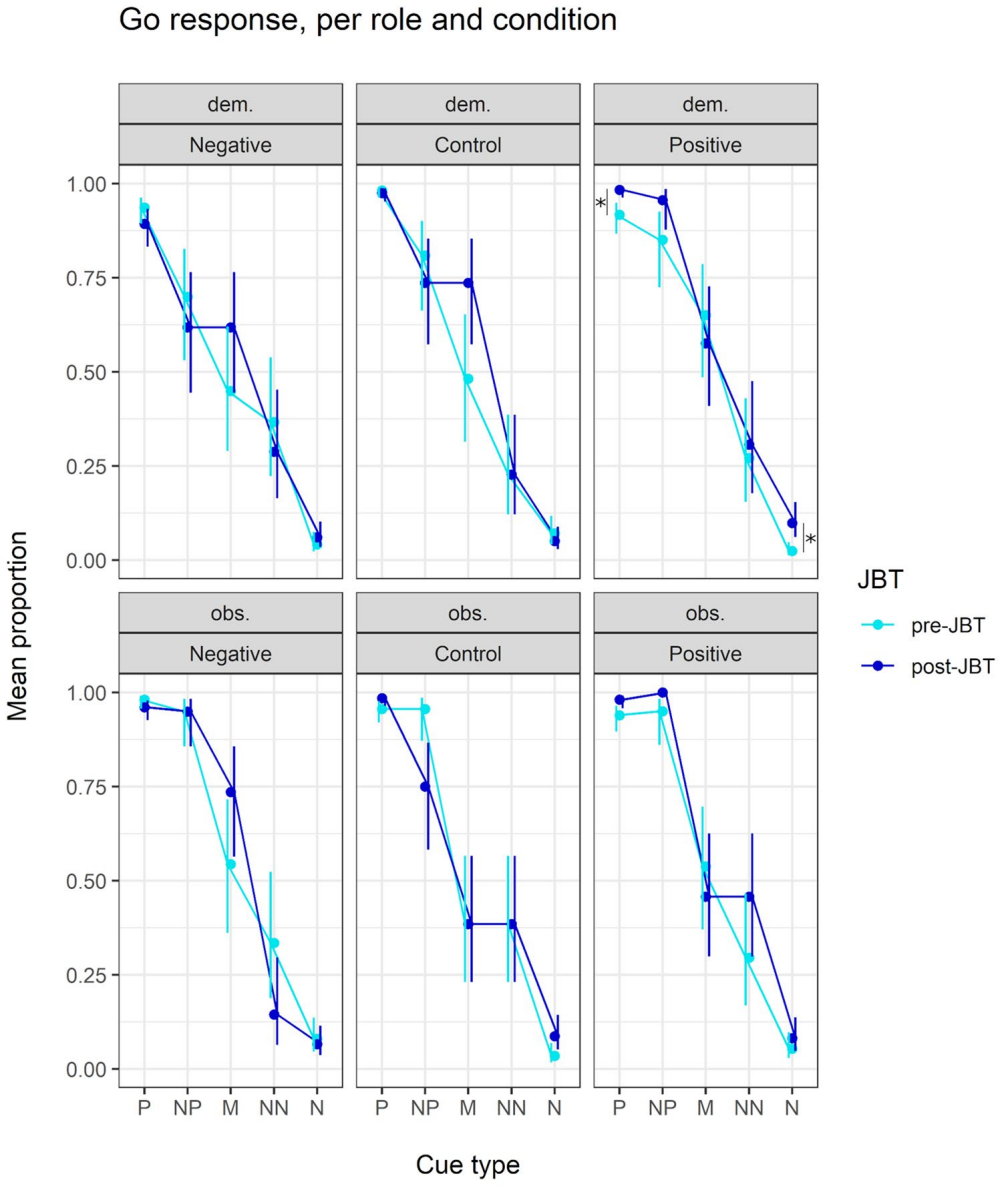
Mean predicted proportion (bars indicate SE) of go response to each of the five cues of the JBT. Plots show go responses between conditions and roles, for each pre- and post-JBT. *P *positive, *NP *near-positive, *M *middle, *NN *near-negative, *N* negative; *dem*. demonstrator, *obs*. observer. *P ≤ 0.05

We predicted that responses to the M cues would show an optimism bias after the positive manipulation, and a pessimism bias after the negative manipulation, for both demonstrators and observers. Our analysis did not support either prediction: After experiencing the positive manipulation, demonstrators did not show a significant increase in go responses to the M cues (compared to pre-test: β =  – 0.318, z =  – 0.398, *P* = 0.691), neither did they show a significant decrease in go responses after the negative manipulation (compared to pre-test: β = 0.685, z = 0.823, *P* = 0.411). Observers did not show a significant increase in go responses to the M cues after positive manipulation to the demonstrator (compared to pre-test: β =  – 0.322, z =  – 0.400, *P* = 0.689), or a significant decrease after the negative manipulation (compared to pre-test: β = 0.846, z = 0.911, *P* = 0.362) (see Fig. 3; and SI for details). Regarding the other ambiguous cues, subjects significantly increased their go response to the NP cue in the post-positive test (compared to post-control: β = 2.711, z = 2.398, *P* = 0.016; to post-negative: β = 2.416, z = 2.127, *P* = 0.034), and more specifically, this concerns the demonstrators (compared to post-negative: β = 2.605, z = 2.145, *P* = 0.032). Though model comparison did not support this interaction effect, we included *role* to further explore our theoretical question of emotional contagion.

#### Responses to the reference cues

We found an unexpected significant effect of condition in response to the reference cues. In the post-positive test, subjects significantly increased their go response to the reference cues *P* and N (compared to pre-positive test, for P: β = 1.452, z = 2.487, *P* = 0.013; for N: β = 1.003, z = 2.102, *P* = 0.036), and in the post-negative test, they significantly decreased their go response to the P cue (compared to post-positive: β =  – 1.481, z =  – 2.507, *P* = 0.012; to post-control: β =  – 1.315, z =  – 2.443, *P* = 0.015). Furthermore, we found that specifically the demonstrators showed an increased response to cues P and N in the post-positive test (compared to pre-positive, for P: β = 1.665, z = 2.052, *P* = 0.040; for N: β = 1.500, z = 2.164, *P* = 0.030), and a decreased response to the P cue in the post-negative test (compared to post-positive: β =  – 1.947, z =  – 2.415, *P* = 0.016; to post-control: β =  – 1.533, z =  – 2.328, *P* = 0.020).

#### Exploratory analyses

For exploratory purposes, we analysed additional factors with a potential impact on the go response. We found a significant main effect of researcher showing overall more go responses when tested by researcher VŠ (compared to JA, β = 0.492, z = 3.146, *P* = 0.002).

### Behaviour during emotion manipulation

#### Negative condition

**Fig. 4:**
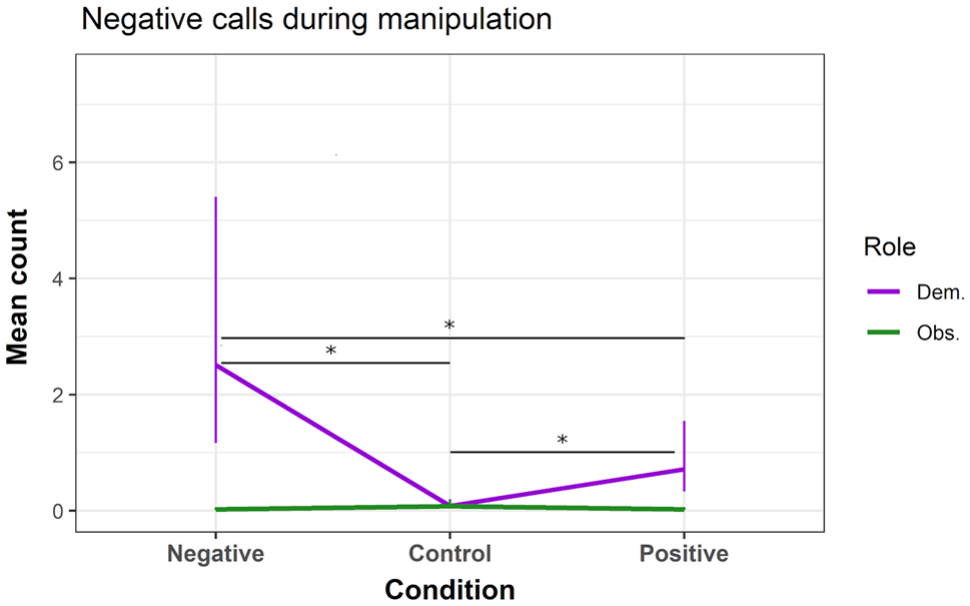
Mean predicted count (bars indicate SE) of negative calls given during the three experimental conditions. *Dem. *demonstrator; *Obs. *observer. *P ≤ 0.001 (note: y-axis scaled to 0–6 for clarification)

**Fig. 5:**
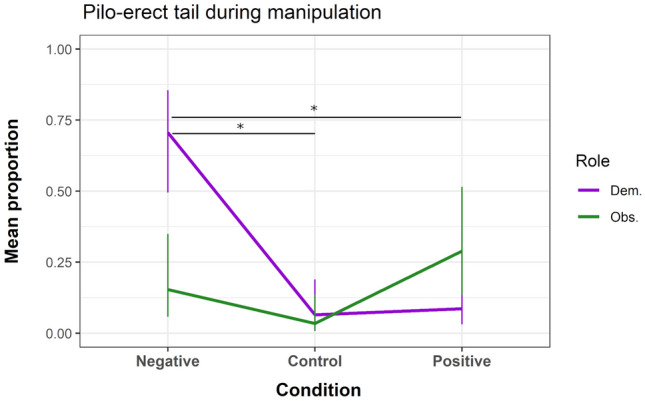
Mean predicted proportion (bars indicate SE) of pilo-erect tail during the three experimental conditions. *Dem. *demonstrator; *Obs *observer. *P ≤ 0.01

As predicted, demonstrators vocalised significantly more negative calls in the negative condition (compared to control condition: β = 3.422, z = 6.228, *P* < 0.001; to positive condition: β = 1.254, z = 6.509, *P* < 0.001; to the observers in the negative condition: β = 4.773, z = 7.809, *P* < 0.001), and unexpectedly, demonstrators gave also significantly more negative calls in the positive condition (compared to control condition: β = 2.167, z = 3.953, *P* < 0.001). Observers showed no significant difference in negative calls between conditions (Fig. 4; see SI for details). Demonstrators also showed significantly more pilo-erected tail in the negative condition (compared to control condition: β = 3.548, z = 2.523, *P* = 0.012; to positive condition: β = 3.242, z = 2.67, *P* = 0.008; to the observers in the negative condition: β = 2.585, z = 2.173, *P* = 0.03). Observers showed no significant difference between conditions (Fig. 5; see SI for details). In contrast to our predictions, demonstrators did not show a significant difference in scratching between conditions (see SI for details). On average, observers scratched significantly more than demonstrators (β = 1.222, z = 2.663, *P* = 0.008), but also showed no significant difference between conditions during the manipulation.

#### Positive condition

**Fig. 6:**
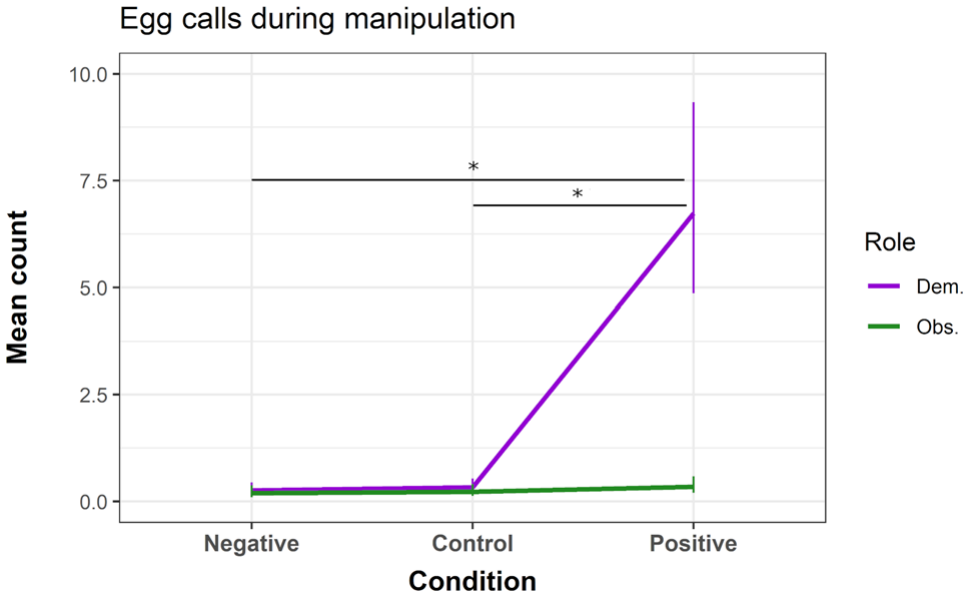
Mean predicted count (bars indicate SE) of egg calls given during the three experimental conditions. *Dem. *demonstrator; *Obs. *observer. * *P* ≤ 0.001

As predicted, demonstrators vocalised significantly more positive calls in the positive condition, compared to the control condition (β = 4.927, z = 5.800, *P* < 0.001) and to the negative condition, where no positive calls were given. Overall, observers emitted fewer positive calls in the positive condition (compared to demonstrators: β =  – 2.671, z =  – 11.402, *P *< 0.001), but they showed no significant difference between conditions (see SI for details). Per exploratory analysis, we found that demonstrators vocalised significantly more egg calls in the positive condition (compared to control condition: β = 3.010, z = 7.915, *P* < 0.001; to negative condition: β = 3.268, z = 6.893, *P* < 0.001; to the observers in the positive condition: β = 2.986, z = 6.579, *P* < 0.001). Observers showed no significant difference in egg calls between conditions (see Fig. 6) (see SI for details). Overall, observers spent more time in front of the box than demonstrators (β = 0.860, *t *= 2.165, *P* = 0.033), but in contrast to our predictions, animals did not spend more time positioned in front in the positive condition (compared to control condition: β = 0.432, *t* = 0.904, *P* = 0.369; to negative condition: β =  – 0.066, *t* =  – 0.138, *P* = 0.891) (see SI for other results).

### Behaviour during JBT

#### Scratching

Overall, subjects showed more scratching in the post-positive test (compared to post-negative: β = 0.666, z = 2.090, *P* = 0.037; compared to pre-positive: β = 0.601, z = 2.016, *P* = 0.044) (see SI Fig. S6). Specifically, observers increased scratching in the post-positive test (compared to the pre-positive: β = 0.847, z = 2.152, *P* = 0.031), and demonstrators decreased scratching in the post-negative test (compared to the pre-negative: β =  – 1.099, z =  – 1.927, *P* = 0.054) (see SI Figs. S7; and SI).

#### Scent-marking

Demonstrators showed significantly less scent-marking in the post-control test (compared to pre-control: β =  – 0.871, z =  – 4.404, *P* < 0.001; compared to post-negative: β =  – 0.562, z =  – 2.560, *P* = 0.010; compared to post-positive: β =  – 0.692, z = 3.346, *P* < 0.001). Observers showed significantly less scent-marking in the post-negative test (compared to post-positive: β =  – 0.551, z =  – 2.937, *P* = 0.003; compared to pre-negative: β =  – 0.524, z =  – 2.796, *P* = 0.005) (see SI Fig. S8; and SI).

#### Gnawing

Demonstrators gnawed significantly less in the post-positive test (compared to pre-positive: β =  – 1.158, z =  – 4.775, *P* < 0.001) and observers gnawed significantly more in the post-positive test (compared to post-negative: β = 0.544, z = 2.711, *P* = 0.006; compared to post-control: β = 0.408, z = 2.008, *P* = 0.044), while gnawing less in the post-negative test (compared to pre-negative: β =  – 0.447, z =  – 2.199, *P* = 0.028) (see SI Fig. S9; and SI).

## Discussion

Our main research question focused on emotional contagion in common marmosets, where we predicted that specific emotional states induced in the demonstrator would transfer to an observer. To this end, we assessed behavioural and cognitive responses, the latter by a judgement bias test. We predicted that demonstrator marmosets exposed to either a positive or negative stimulus would show an optimism or pessimism bias, respectively, in their responses to the ambiguous middle cue in the post-JBT. Additionally, we predicted that an induced positive state would correlate with emitting positive calls and by bodily positioning in front of the shown stimulus, and that an induced negative state would correlate with emitting negative calls, and showing more pilo-erected tail, scratching, and less positioning in front of the stimulus. We further investigated emotional contagion in the observer, and predicted to find an optimism or pessimism bias in the positive or negative condition, respectively. Moreover, we predicted positive or negative state-related behaviours, similar to the expressions seen in the demonstrator. Although our emotion induction in the demonstrator seemed successful, at least based on the behavioural assessment, our emotional contagion hypothesis was not confirmed, and we did not find the predicted state-related behaviours in the observer, though we did find unexpected behaviour in the post-positive condition. Moreover, neither animal showed an optimism or pessimism bias for the middle ambiguous cue in the post-JBT, yet further analyses provided also here unexpected response changes to other ambiguous and reference cues. We will first discuss the cue responses in the JBT, followed by a discussion of the behaviour shown during emotion manipulation, and lastly behaviour shown during the JBT.

### Cue responses in the JBT

To verify the JBT paradigm, we performed two manipulation checks, namely discrimination training success and order effect of the ambiguous cues. Subjects showed successful discrimination between the reference P and N cues, including an intermediate valuation of the NP, M, and NN cues, resulting in a typical monotonically graded response curve (Gygax 2014). Subjects also showed no-learning effect of the NP, M, or NN cues, a necessary prerequisite for further interpretation of the test results. Though our choice for rewarding the ambiguous cues (in line with Hintze et al. 2018) did not induce a learning effect, it might be interesting to consider an intermediate reward schedule for future studies. Nevertheless, despite a successful training and no-learning effect, and a potential successful emotion manipulation in the demonstrator, the manipulation may not have been strong enough to either temporally last until, or be detected by, the post-JBT. Accordingly, we found no congruent optimism or pessimism bias in the response to the ambiguous M cue, in either positive or negative conditions. Interestingly, demonstrators showed an increased go response to the NP cue in the post-positive JBT, as well as to the reference P and N cues, while showing a decrease in go response to the P cue in the post-negative JBT. In addition, our study showed a researcher-dependent effect on the JBT, with a higher go response when tested with one of the two researchers involved in data collection.

A seemingly pivotal element of a JBT to define anticipation of a positive or negative event, is the middle ambiguous cue, as this cue is truly ambiguous, and thus, emphasizes the uncertainty during decision-making. However, as noted in a recent meta-analysis (Lagisz et al. 2020), the most ambiguous cue does not necessarily evoke the greatest response change, and it is greatly recommended to apply a multiple cue design. Moreover, a response change to the near-reference cues NN and NP may indicate specific biases that allow to disentangle same-valence states. For instance, a decreased response to NN may reveal an increased expectation of negative events (i.e., anxious state), and a decreased response to NP may depict a decreased expectation of positive events (i.e., depressive state) (Mendl et al. 2009). Similarly, an increased response to NP could reveal an increased anticipation of a positive event. This interpretation is in line with demonstrators showing more approach to NP after the positive condition, supporting the cognitive bias hypothesis, and providing evidence for a positive emotional state as detected by the JBT. However, considering that our results also show response changes toward the reference cues, we are inclined to approach this interpretation with caution. These changes put into question the validity of our task design and the interpretation of other JBT results, such as to the NP cue. Generally, no bias effects are expected at the reference cues, as the reward values of these cues have been established during training, and therefore have more certainty than the ambiguous cues (Gygax 2014; Neville et al. 2020). Still, some studies have reported effects at the reference cues as well (Lagisz et al. 2020), with in particular changed responses to the N cue (Neville et al. 2020). It is suggested that effects at the reference cues are either due to ineffective training (e.g., due to the task being too difficult, e.g., Bateson et al. 2011), due to an intense affect manipulation, or due to interference with the emotion manipulation (Lagisz et al. 2020). Considering our strict training criterion following previously established paradigms, and the JBT discriminatory success during testing as supported by the monotonic graded response curve, the effects found on the P and N cues are more likely to have happened due to potential conflict with our emotion manipulation. Indeed, this is supported by the finding that specifically demonstrators changed their response to the P and N cues, as these subjects were directly exposed to the food reward in the positive condition. Potentially, the use of food, rather than non-food, as positive stimulus, altered the demonstrators perceived reward value of P and N, and this potential confound should be considered in further research. Alternatively, a general increase in motivation to perform could have driven these results, yet this should have also prompted more responses to the ambiguous M and NN cues, which was not the case. Therefore, it seems that there is a specific change potentially due to used food reward for the positive manipulation. This pattern is perhaps also observed in the effects of researcher identity. Here, the higher response potentially indicates a change in reward expectation, where a higher reward is anticipated when the test is conducted by VŠ. We assume that this is due to researcher VŠ having worked in the marmoset lab for more years than researcher JA, in a variety of experimental set-ups that included food rewards. Despite our extensive efforts to standardize the protocol, in which both researchers were trained to operate the apparatus and to interact with the subjects identically, it seems the more familiar researcher had a stronger reward expectation effect on the subjects, which was then detectable through the judgement bias test. Another study on marmosets investigated researcher identity and found that it may impact participation, but not performance (Schubiger et al. 2015), and we are unaware of any judgement bias study showing a researcher effect. To conclude, these results of response changes to other cues, rather than solely to the middle ambiguous cue, highlight the importance of including all cues to the statistical analyses (Gygax 2014), as well as adding the full dataset with other, potentially important variables such as researcher identity, to increase the analyses’ power (Lagisz et al. 2020).

In recent years, the use of the cognitive bias paradigm, and specifically the judgement bias design, has risen in popularity, resulting in a plethora of studies applying the test. Nevertheless, several reviews have raised important concerns regarding methodological and theoretical questions (Mendl et al. 2009; Bethell et al. 2015; Roelofs et al. 2016). A first meta-analysis concluded that, when controlling for potential drug side-effects, the judgement bias paradigm is a valid measure to assess the positive or negative association of pharmacologically induced states in animals (Neville et al. 2020). A second meta-analysis focussing on non-pharmacological affect manipulations (Lagisz et al. 2020) also found general support for the judgement bias paradigm as a valid measurement of affect in animals. Importantly, the authors emphasize the need for more and continued validation of the paradigm, as there is great variability in effect sizes between studies and in the extent that experimental design details are reported, as well as the need for more empirical research in regard to different design types, including species-relevant set-ups and cues. Indeed, in the human emotion field, where the cognitive bias hypothesis originates from, investigation of the paradigm is ongoing to understand all different aspects of the decision-making process and how affect may play a role (Iigaya et al. 2016). In this vein, there are very few studies on the use of the JBT in primates, which is potentially indicative of (methodological) difficulties inherent to the bias paradigm’s requirements. In particular for the primate group, more empirical research is thus required to validate the paradigm.

Furthermore, these results might also, or in addition, be due to a lack of manipulation effect, and specifically, the JBT could have benefitted from a more realistic intervention rather than a static one (e.g., a simulation procedure, as in Adriaense et al.  2019a). Still, other studies have successfully used artificial toy predators as manipulation (see Neal and Caine 2016, for examples). Moreover, most studies on cognitive bias focus on long-term moods through environmental changes (e.g., enrichment in pigs, Douglas et al. 2012) or social behaviours (e.g., long-term grooming in Schino et al. 2016), but effects of short-term social behaviour may show different results (e.g., immediate grooming showed no bias, in Schino et al. 2016; though see e.g., Rygula et al., 2012; Adriaense et al. 2019a, for short-term effects). Others have also raised concerns regarding the success rate of affect induction in cognitive bias tests, calling for further validation of its paradigm (Košťál et al. 2020).

### Behaviour during emotion manipulation

As predicted, during the positive manipulation, demonstrators gave more positive calls, and during the negative manipulation, they emitted more negative calls and showed pilo-erected tail. The observation that types of calls given by the demonstrator reflect the positive or negative condition, is congruent with the previous findings of these calls in contexts of either food anticipation or high vigilance and predator mobbing (e.g., Epple 1968). Furthermore, pilo-erected tail is often used as behavioural indicator of arousal (Schubiger et al. 2015), and is also observed in negative-related conditions (Ermatinger et al. 2019). This is consistent with the observation of increased pilo-erected tail in our negative condition, and together with the negative calls, this indicates a negative, aroused state in the demonstrator. Interestingly, demonstrators showed no distinction in terms of staying in front of the stimulus instead of elsewhere in the experimental cage. This latter result calls into question the fear-inducing aspect of the negative manipulation, as in a fear context we would expect an avoidance rather than an approach response. Furthermore, during the positive manipulation, demonstrators also gave negative calls (i.e., as compared to control), and more egg calls. Independent egg calls are often observed when facing a threat or aggression (Bezerra and Souto 2008; Epple 1968) and, thus, probably reflect vigilance and negative context. We are unaware of studies observing egg calls in positive contexts, though a food context may not necessarily be positive as it may elicit food competition, which is potentially stress-inducing (e.g., Tardif and Richter 1981, see discussion below). Additionally, a food context may lead to frustration in the demonstrator because the food is inaccessible, and as such, the negative and egg calls may reflect frustration. This shift from positive anticipation to frustration is often discussed in emotion research (Briefer et al. 2015; also mentioned as argument for the positive condition in Adriaense et al. 2019a). In this case, it could be that the observer picked up this state in the demonstrator, and subsequently showed congruent frustration-related scratching in the post-positive JBT. Despite that the other predicted behaviours of staying in front of the stimulus and scratching during the manipulations were not supported in our sample, the remaining observed behaviours provide evidence for a distinction between the two manipulations. Therefore, in conclusion, demonstrators show distinct behaviours between the two manipulations, with contrasting vocalisations and pilo-erected tail, confirming their general positive and negative inducing effect. Yet, the demonstrator’s response to stay in front of the negative stimulus, rather than moving away, potentially warrants against a more precise interpretation and raises the question whether the manipulation indeed induced a fear-related state. Furthermore, and upon seeing the demonstrators, the observers showed no predicted or manipulation-related behaviour. Therefore, based on the observable behavioural expressions of the observers during manipulation, we cannot conclude that emotional contagion occurred. Future research could make use of thermography as a more fine-grained method to measure emotional arousal, certainly in an emotional contagion context (Ermatinger et al. 2019; Brügger et al. 2021).

### Behaviour during JBT

To further assess the emotion manipulation effect, we analysed behavioural responses during JBT as well. Due to the subjects’ simultaneous occupation with the JBT, the overall frequencies of these additional behaviours were low, yet they may help to clarify whether the manipulation was either not strong enough to last until, or be detected by, the JBT. Results show that after the positive manipulation, demonstrators decreased gnawing, and observers increased scratching, as well as scent-marking and gnawing (i.e., latter two from between-condition comparisons). After the negative manipulation, demonstrators decreased scratching, and observers decreased scent-marking and gnawing. The significance of each of these behaviours and their changes is challenging to interpret without other specific measurements, and our results from the JBT do not facilitate interpretation. Still, each of these behaviours has been studied in relation to a variety of social contexts in other studies and we will discuss our results in light of these.

### Scratching

Scratching is commonly observed in primates in negative situations, for instance in occurrences of social conflict (Aureli and van Schaik 1991), contradicting motivations (Troisi et al. 1991), or predatory threat (see Neal and Caine 2016, for overview). Depending on the specific circumstances, scratching may thus reflect negative stress or anxiety, and therefore, scratching has been suggested as general indicator of a negative emotional state (Maestripieri et al. 1992; Troisi 2002). In marmosets, this is supported by observations of increased scratching during mildly stressful husbandry procedures (Bassett et al. 2003), and decreased scratching when animals are given anxiety-reducing drugs (Cilia and Piper 1997) or after positive interaction with human caretakers (Manciocco et al. 2009). In our study, subjects showed an increase in scratching in the post-positive test, and specifically, demonstrators decreased scratching in the post-negative test, and observers increased scratching in the post-positive test. This result is in contrast to our prediction, as we expected to find more scratching in the negative condition, both during manipulation and during post-JBT. The decreased scratching in the demonstrator may perhaps support the interpretation that, despite the negative manipulation apparently being successful, the effect was not strong enough to last until the post-JBT. However, demonstrators did not increase scratching during the negative manipulation and, moreover, observers showed an increase in scratching in the post-positive JBT. Therefore, we suggest that scratching in this study, particularly in the post-positive test, may not necessarily reflect anxiety, but perhaps indicates a negative state similar to frustration or conflicting motivations. Indeed, scratching has been proposed to reflect mild anxiety, yet with increasing anxiety the relation with scratching follows an inverted U-shape (Troisi et al. 1991). Emotional contagion has been suggested to facilitate a variety of social behaviours, such as food competition, in which an initially assumed positive context, and perhaps state, changes into a more negatively associated context, and thus, potentially also negative state. Our demonstrator-observer design does not exclude these other social elements. Seeing a group mate in a seemingly positive state may put the observer in a conflicting state due to not being able to get the same context as the demonstrator. This seems a plausible hypothesis as watching the other group member in a beneficial context may induce food competition, which is known to be stress-inducing (e.g., Clay and de Waal 2015; Tardif and Richter 1981). This is further supported by findings of a recent thermography study (Ermatinger et al. 2019), suggesting that preferred food may induce food competition related to negative arousal. For that reason, the increased scratching in the post-positive JBT may reflect a negative state in the observer.

### Scent-marking and gnawing

Scent-marking is often observed when common marmosets are in new environments (Epple 1970) and functionally serves territorial defence and reproductive status advertisement (Harrison and Tardif 1988; Lazaro-Perea et al. 1999). In our study only social group members took part in the experiment, which excludes territorial defence, and the composition of pairs did not include any adult female–male pairs, which excludes status signalling. Furthermore, marmosets usually gnaw holes in trees to extract gum, which is a behaviour often observed as fixed action pattern with scent-marking after gnawing is completed (Lazaro-Perea, 1999; Massen et al. 2016). Interestingly, scent-marking and gnawing are suggested to be arousal-related behaviours (e.g., both are part of the arousal cluster in Martin et al. 2019; scent-marking is part of the stress-activity cluster in Šlipogor et al. 2016; 2021). In that vein, our data may indicate that the different manipulations were not sufficiently arousal-inducing to have a lasting temporal effect until the post-JBT. During the negative manipulation, demonstrators showed more pilo-erected tail, and thus, it could be expected that this greater arousal would be reflected in the other behaviours during JBT. Yet, demonstrators showed no change in scent-marking in either post-positive or -negative JBT, and even showed a decrease in gnawing after the positive manipulation. Interestingly, observers exhibited a condition-dependent pattern in which more scent-marking and gnawing occurred in the post-positive JBT, and less scent-marking and gnawing in the post-negative JBT. If these behaviours are indeed related to arousal, then this indicates that watching the demonstrator specifically during the positive manipulation was more arousal-inducing for the observers than the negative manipulation. Interestingly, some have suggested that scent-marking may be a (negative) stress-related behaviour, though perhaps less sensitive than scratching (Cilia and Piper 1997; Bassett et al. 2003). In regard to our results, this would indicate that watching the demonstrator in a positive food context induces both a higher arousal and negative state in the observer.

### Emotional contagion?

When combining all results of the JBT and the behaviours during manipulation and JBTs, we find further support for our *post hoc* food competition hypothesis. In the post-positive condition, observers showed more scent-marking and gnawing than in the post-negative condition, which may reflect high arousal and to some extent negative stress, while also displaying more scratching, which is assumed to indicate negative affect. This combination suggests that watching the demonstrator in the positive condition generated a context of high arousal and negative affect for the observer. Perhaps our specific design prompted an unintended food competition, where the observer was not able to directly experience the positive manipulation itself, yet picks up on the food cues from the demonstrator. This interpretation is consistent with the finding of positive food calls and egg calls in the demonstrator in the positive condition, and also to some extent negative calls, and these calls combined may indicate a positive, but also a vigilant state. Thus, these calls could be indicative of a competitive setting. Our argument of competition in the positive condition, rather than strict emotional contagion, is consistent with the post-negative results, showing decreased arousal-related behaviours in the observer, indicating that watching the demonstrators here was not arousal-inducing. The competition hypothesis is further supported by reduced scent-marking in the observer and the lower rates of scratching in the demonstrator, which both may reflect the absence of a negative state in the post-negative condition. Moreover, the judgement bias results provide additional tentative support, as demonstrators showed increased responses for the near-reference NP and reference P and N cues in the post-positive manipulation. As discussed previously, this may be indicative of a higher reward expectancy, and if indeed the increased responses to NP, P, and N reflect this, then this confirms the efficacy of the positive manipulation effect, which then again may explain the observer’s behaviour in the post-positive condition. However, it is important to point out that the response to the reference cues ideally remains unchanged, though studies have reported different results, as discussed previously.

Importantly, a small number of studies report that scratching as indicator of a negative state is perhaps not as empirically supported as initially assumed (see Neal and Caine 2016, for overview). For instance, Barbary macaques show increased scratching after (assumingly positive) grooming bouts, which potentially puts the assumed negative valence of scratching into question (see Semple et al. 2013 for discussion, but see Ueno et al. 2015, for decreased scratching after grooming; see Berthier et al. 2018, for decreased scratching after observing others groom). Studies in common marmosets find that subjects undergoing anxiety-inducing manipulations of social isolation, food competition, and predatory threat show a decrease in scratching *during* these manipulations (Neal and Caine 2016). Moreover, *after* manipulations of social isolation, predatory threat, and administration of anxiogenic drugs, marmosets do not increase scratching (Kato et al. 2014; but see Cilia and Piper 1997). Accordingly, researchers have called for awareness about the assumed emotional state underlying scratching, thereby suggesting that scratching may be associated with a general arousal level, or even positive arousal, depending on the context (Neal and Caine 2016). Though a direct examination of the correlation between scratching and positive arousal is missing in the current research (Neal and Caine 2016), it remains an interesting notion in light of our study. It is possible that the observer’s scratching relates to socio-positive behaviour reminiscent of positive excitement. Watching the demonstrator in a rewarding or beneficial context may induce a positive state, because that reward may eventually be beneficial to the observer, or may induce a general positive affect (Nakahashi and Ohtsuki 2018). The latter notion is supported by the evidence that common marmosets are highly prosocial (Burkart et al. 2007, 2014), and thus, observers may experience seeing the other in a beneficial context as rewarding to themselves. Neal and Caine (2016) conclude that scratching has perhaps been too easily assumed to be negative, and accordingly, a priori expecting an all-or-nothing relation between negative events and scratching may have unwanted consequences to the progress of this research topic, as it may result in a lack of alternative explanations.

The potential presence of a competitive context also highlights the difficulty of investigating emotional contagion. The concept itself may underpin a variety of social behaviours (e.g., predator mobbing or conflict management) which result in various combinations of similar and/or differing emotional states (e.g., a matching state may be counterproductive in situations of consolation or helping, Adriaense et al. 2020), which additionally depends on the specific actors and context (Dezecache et al. 2015). This may explain the relatively low number of experimental studies on emotional contagion, despite its popular status due to its relevance for empathy, and despite the growing interest in animal emotions in social settings such as in animal welfare (Baciadonna et al. 2018). Moreover, the study of emotions and their induction in a laboratory setting is challenging, even in humans, and, for instance, positive or low arousal states remain particularly difficult to assess in animals (Mendl et al. 2009). This may explain why the research field has been primarily dominated by research on high arousal, and intense negative states such as pain (Boissy 2007; Meyza et al. 2017). In that vein, we recommend for future research to further explore different arousal and valence states, as well as aiming to disentangle emotional contagion effects from other social effects, such as the mere presence of a demonstrator partner by using an additional control condition without the demonstrator present.

## Conclusion

Although common marmosets’ social lifestyle in extended family groups demands social skills, including the need for efficient communication and coordination, we did not find evidence of emotional contagion through a judgement bias paradigm in this study. Yet, it is unclear whether this was due to our study design and its specific emotion manipulation, or a general absence of emotional contagion in common marmosets. Based on some of the behavioural parameters, the demonstrators’ emotional states were seemingly successfully manipulated in the study. We found no expected response change to the middle ambiguous cue, but we found an increased response to the NP cue in the post-positive-JBT, yet, as we also found responses changes to the references cues, the NP result warrants caution. As JBTs usually assess mood, they may be less sensitive to detect affective changes in short-term manipulation designs. Therefore, more empirical research on the relation between long- and short-term social behaviours and judgement bias is needed, as well as a better understanding of different emotion manipulations and potential external effects leading to measurement bias such as researcher identity. Further, we found an interesting combination of increased scratching, scent-marking, and gnawing in the observer after watching the demonstrator undergoing a positive manipulation. Nevertheless, it remains unclear how to interpret these particular condition-dependent changes. This difficulty to infer the specific emotional states relevant to our contrasting hypotheses (i.e., emotional contagion and food competition) underlines one of our main arguments on measuring animal emotions, which is that without additional objective investigation, it remains particularly challenging to interpret valence from overt, behavioural observations. Therefore, further research is required to explore our *post hoc* food competition hypothesis, and to verify the presumed state of our measured behaviours in different contexts. Importantly, future work should consider the facilitating effect of emotional contagion, and aim at more precisely analysing its information transmission function.

## Supplementary Information

Below is the link to the electronic supplementary material.

Supplementary file1 (XLSX 182 KB)

Supplementary file2 (DOCX 28 KB)

Supplementary file3 (DOCX 1620 KB)

Supplementary file4 (DOCX 22 KB)
