# Supplementary material for: Watching others in a positive state does not induce optimism bias in common marmosets (Callithrix jacchus), but leads to behaviour indicative of competition
Source: Anim Cogn. 2021 Mar 16;24(5):1039–56. doi: 10.1007/s10071-021-01497-1 (PMC8360889; doi:10.1007/s10071-021-01497-1)
Supplement: Supplementary file 2 — Supplementary file2 (DOCX 28 KB) [file 10071_2021_1497_MOESM2_ESM.docx]

**Supplementary Info -** *Watching others in a positive state does not induce optimism bias in common marmosets (Callithrix jacchus), but leads to behaviour indicative of competition*. Adriaense J.E.C., Šlipogor V., Hintze S., Marshall L., Lamm C., Bugnyar T.

1. **Methods**

**Housing**

The 8 subjects were housed in four separate social groups (Group 1: Aurora; Group 2: Oli and Luna; Group 3: Fimo and Locri; Group 4: Smart, Simba, and Nala). Group 2 and Group 4 were housed with other family members, including a breeding pair and their offspring, Group 1 was housed with other unrelated conspecifics, and Group 3 were the only members of this social group. The four groups lived within acoustic and olfactory range from each other. Each group was housed in wire mesh indoor home enclosures (approx. 250 x 250 x 250 cm), with access to outdoor enclosures (approx. 250 x 250 x 250 cm) and to the experimental enclosures through a tunnel system. Animals had free access to both indoor and outdoor enclosures during warmer periods of the year, and outdoor access was restricted in winter, when outside temperature fell below 5°C. The rooms had windows for natural light, indoor temperature was kept between 21°C and 29°C, humidity between 30% and 60%, and additional lamps provided a 12:12 h light:dark cycle. Heating lamps were provided to optimize their well-being. Every home enclosure consisted of various enrichment objects (branches, ropes, platforms, blankets, sleeping baskets) with wood pellets as floor bedding. All animals were fed twice daily (i.e. around 7:00 and around 12:00) with a varied diet of marmoset pellets and jelly, fruits, vegetables, grains, milk products, protein and vitamin supplements, and insects. Water was available *ad libitum* for each group. The housing conditions were in accordance with institutional guidelines, Austrian legislation, and the European Association of Zoos and Aquaria husbandry guidelines for Callitrichidae.

**Dyads**

Dyads were formed with an individual from the same housing group, making for a total of 5 dyads (Luna & Oli, Fimo & Locri, Smart & Nala, Smart & Simba, Nala & Simba). Each subject took on the role of demonstrator and observer min. once and max. twice. One subject (Aurora) was tested as single demonstrator throughout the entire study as her designated partners did not reach training criterion within the set time period (we decided to not exclude her to increase sample size for verifying the emotion manipulation).

**Habituation**

Judgement bias testing is often done in isolation, rather than in the animal’s social group, and this may increase stress and decrease willingness to participate in the experimental testing (Roelofs et al., 2016). We incorporated an extensive habituation protocol for all tested subjects to enter and stay in experimental cages alone, the training was done in the same location as testing, and overall subjects were already familiar with this location due to participation in previous studies. At all times, the subjects’ family group members remained in the tunnels in the experimental room and were within auditory reach.

**Apparatus training**

Apparatus training consisted of **initiator and door training**. During **initiator training**, we trained subjects to touch a trial initiator (i.e. white plastic object, 20 x 4 x 1 cm), by holding the initiator in one hand and rewarding the subject with the other hand, after which we gradually increased the spatial distance between touching and receiving reward. The end result of this training was achieved when subjects touched the trial initiator and then crossed the cage to its opposite side to receive a reward (total distance: 100 cm). The trial initiator was then attached to a string, which could be manipulated by a researcher standing on the other side of the cage (see SI Figures S1, S2, and S3). Here we again trained subjects to touch the trial initiator, after which it was pulled up and out of view of the subject, and then subjects were expected to cross the cage over to the researcher to receive a reward.

This training was followed by **door training**, which refers to the five doors of the JBT apparatus (see SI Figure S5). Here, subjects were trained to touch the trial initiator, then cross the cage and sit in front of the door that was opened by the researcher. This training was done to ensure subjects kept using the trial initiator to actively start the next trial and both doors (i.e. reference P and N cue) were equally rewarded to ensure attention to both cues. Some of the criticism of the judgement bias paradigm is the potential issue that animals learn to neglect the N cues, as these are often unrewarded from the start of the discrimination training (also noted by Hintze et al., 2018). Such learned disregard for certain cues and their related cue characteristics (e.g. spatial orientation), may lead to disregard of other similar cues later during testing. Concretely, the most left or right cue is trained as the N cue, and when animals learn to avoid N, they may apply a strategy to visually or auditorily neglect this side of the spatial test. During testing the additional NN, or even M, cues may also be ignored as they visually are fully, or partially, in the disregarded area. A potentially found pessimism bias may thus be due to disregard of these cues, rather than a negative judgement of their ambiguity. To counter this potential neglect, we followed the training schedule by Hintze et al. (2018). Subjects were first presented with 20 rewarded N trials and after reaching training criterion, the N trials remained unrewarded (criterion was set at 80% go response to 15 P and 15 N cues, within 10 s of touching trial initiator, see SI Table S4 for details). In addition, when a cue door opened, both a visual element as well as an auditory one was present. This way, even if animals did not immediately look at the opened door, the auditory element added a second salient effect to increase their attention.

1. **Testing procedure**

Between each test session all three compartments were cleaned with a vinegar-water solution. Trials were presented in semi-random order with the first trial always being P, and NP, M, and NN followed P or N in a balanced order between test sessions. One JBT consisted of 13 trials (i.e. 5 P; 1 NP; 1 M; 1 NN; 5 N) and per test session two JBTs were conducted per subject (i.e. one pre- and one post-JBT), resulting in 26 trials per session (i.e. 20 P; 2 NP, 2 M, 2 NP, and 20 N). This gives in total for period 1 12 trials for each ambiguous cue per subject, and thus 24 trials after repeated measures and in line previous studies (e.g. Pomerantz et al., 2012; Bateson and Nettle, 2015; Bethell et al., 2016).

**Welfare**

To ensure subjects’ wellbeing, we only continued testing when the animals showed no overt signs of distress. We terminated the session if we observed any of the following: repeatedly moving back and forth to the compartment’s exit door, remaining at the exit door (for longer than 10 s), staying in one location of the compartment without moving (for longer than 15 minutes), quickly jumping back and forth in the compartment with jerky movements and/or emitting lots of ‘phee’ calls.

1. **Results**

See our Excel data file “Adriaense et al., 2020. Anim Cog – datafile” for all raw data used in the statistical analyses.

**Variables**

The following variables were either predicted or used for exploratory purpose. All were used for model comparison with AIC, and depending on the likelihood ratio rest and AIC value, the variables were used for further analyses.

The response or dependent variables were: go response (yes, no), behaviour during JBT (scratching, scent marking, gnawing), behaviour during emotion manipulation (negative calls, positive calls, egg calls, contact calls, pilo-erect tail, scratching, scent marking, gnawing, position).

Predicting or independent variables were: researcher (VŠ, JA), role (demonstrator, observer), period (1 or 2), condition (positive, negative, control), cue (P, NP, M, NN, N), test (pre-JBT, post-JBT), subject (Aurora, Luna, Oli, Fimo, Locri, Smart, Nala, Simba), date (date of testing), and time (AM, PM).

**Cue responses during JBT: additional results**

We verified whether the go response to the M cue would change between pre- and post-JBT, within the different conditions, and for each role (which we added in interaction for theoretical reasons). For this we used a model including *researcher* and *period* as main effects, and *role/condition/cue/test* as interaction effect. Additional results showed that there was no change in response to the M cues in the post-JBT of the control condition (compared to pre-control, for demonstrators: β = 1.100, z = 1.267, P = 0.205; for observers: β = 0.000, z = -0.000, P = 1). Results also showed that over time, subjects significantly decreased their go response to all cues (compared to period 1: β = -0.603, z = -3.267, P = 0.001).

**Behaviour during manipulation: model comparison**

Based on a likelihood ratio test, comparing the basic model with a full model, and the AIC, the best model for **negative vocalizations** included *time* as predictor; for **positive vocalizations** *time* (in the observer subset) and *period* (in the demonstrator subset); for **egg calls** *period* as main effect and *role/condition* as interaction effect; for **contact calls** *role/condition* as interaction effect; for **pilo-erect tail** *time* as main effect and *role/condition* as interaction; for **scratching** *role* as main effect; for **scent-marking** *period* as main effect and *role/condition* as interaction effect; for **gnawing** *period* as main effect; and for **front position** *role* as main effect. Each of these models included *subject* and *date* as random variables, except front position with only *subject* as random variable.

**Behaviour during manipulation: additional results**

*Negative calls*

Overall, marmosets showed a significant small increase of negative calls when tested in the afternoon (β = 0.455, z = 2.151, P = 0.032). Demonstrators gave significantly more negative calls in the positive condition, compared to the observer (β = 3.343, z = 6.400, P < 0.001). Observers showed no significant difference in negative calls between conditions (between negative and control condition: β = 1.264, z = 1.593, P = 0.111; between negative and positive condition: β = 0.176, z = 0.239, P = 0.811).

*Positive calls*

As demonstrators showed no positive calls in the negative condition, we subset the data by *role* (dataset 1: demonstrators, positive and control; dataset 2: observers, all conditions). Over time, demonstrators showed a significant decrease in positive calls (compared to period 1: β = -2.406, z = -2.563, P = 0.010). Observers showed a significant decrease in positive calls when tested in the afternoon (β = 2.148, z = 2.239, P = 0.025), and showed no significant difference between conditions (between negative and control condition: β = -0.930, z = -1,070, P = 0.285; between negative and positive condition: β = -0.654, z = -0.786, P = 0.432*).*

*Egg calls*

Over time, subjects showed a significant decrease in egg calls (compared to period 1: β = -0.704, z = -2.595, P = 0.009). Observers showed no significant difference in egg calls between conditions (between positive and control condition: β = -0.400, z = -0.673, P = 0.552; between positive and negative condition: β = -0.571, z = -0.782, P = 0.434).

*Pilo-erect tail*

Overall, marmosets showed a significant increase of pilo-erect tail when tested in the afternoon (β = 2.001, z = -2.557, P = 0.011). Observers showed no significant difference in pilo-erect tail between conditions (between negative and control condition: β = -1.639, z = -1.026, P = 0.305; between negative and positive condition: β = 0.807, z = 0.706, P = 0.480).

*Scratching*

Demonstrators showed no significant increase in scratching in the negative condition (compared to control condition: β = 0.419, z = 0.445, P = 0.656; to positive condition: β = 0.397, z = 0.428, P = 0.669).

*Additional exploratory analyses*

Demonstrators gave more contact calls (i.e. phee, shrill, and whirr) in the positive condition (compared to the negative condition: β = 0.894, z = 3.626, P < 0.001) and in the control condition (compared to the negative condition: β = 0.669, z = 2.239, P =0.025). Observers emitted less contact calls in the positive condition (compared to the negative condition: β = -0.470, z = -2.049, P < 0.001), and when compared to demonstrators in the negative condition, observers gave more contact calls (β = 0.998, z = 3.870, P < 0.001). Furthermore, all subjects showed a significant increase of scent-marking over time (compared to period 1: β = 1.741, z = 3.355, P < 0.001), and demonstrators showed more scent-marking in the control condition (compared to the positive condition: β = 1.387, z = 2.338, P = 0.019). Over time, subjects also showed a significant increase in gnawing (compared to period 1: β = 2.641, z = 2.310, P = 0.021). Subjects only occasionally showed self-grooming, defecation, and urinating, so no further analyses were performed for these behaviours.

**Behaviour during JBT: model comparison**

The best model for **scratching** included *time* as main effect, *condition/test* as interaction effect, and *subject* as random variable; for **scent-marking** and for **gnawing** each model included *period* as main effect and *role/condition/test* as interaction.

**Behaviour during JBT: additional results**

*Scratching*

On average, subjects scratched significantly less when tested in the afternoon (compared to the morning: β = -0.469, z = -2.301, P = 0.021). All subjects showed less scratching in the pre-control test (compared to the pre-negative: β = -0.934, z = -2.273, P = 0.023; post-control: β = -0.965, z = -2.351, P = 0.019). After adding *role* as interaction effect, we found that observers increased scratching in the post-control test (compared to the pre-control: β = 1.299, z = 2.017, P = 0.044).

*Scent-marking*

Over time, subjects showed a significant small increase in scent marking (compared to period 1: β = 0.252, z = 3.069, P = 0.002).

*Gnawing*

Overall, subjects showed more gnawing in period 2 (compared to period 1: β = 0.809, z = 5.453, P < 0.001). Demonstrators gnawed more in the pre-positive test (compared to pre-negative: β = 0.546, z = 2.502, P = 0.012) and more in the pre-control test (compared to post-control: β = 1.059, z = 3.794, P < 0.001). Observers also showed a significantly higher count of gnawing in all post-tests (compared to demonstrator data, for post-negative: β = 0.876, z = 3.195, P = 0.001; for post-positive: β = 1.596, z = 6.653, P < 0.001; for post-control: β = 1.434, z = 5.207, P < 0.001).
