# Supplementary material for: Watching others in a positive state does not induce optimism bias in common marmosets (Callithrix jacchus), but leads to behaviour indicative of competition
Source: Anim Cogn. 2021 Mar 16;24(5):1039–56. doi: 10.1007/s10071-021-01497-1 (PMC8360889; doi:10.1007/s10071-021-01497-1)
Supplement: Supplementary file 3 — Supplementary file3 (DOCX 1620 KB) [file 10071_2021_1497_MOESM3_ESM.docx]

**Supplementary Figures -** *Watching others in a positive state does not induce optimism bias in common marmosets (Callithrix jacchus), but leads to behaviour indicative of competition.* Adriaense J.E.C., Šlipogor V., Hintze S., Marshall L., Lamm C., Bugnyar T.

- S1. Experimental design
- S2. Front view demonstrator’s compartment
- S3. Side view demonstrator’s compartment
- S4. Stimulus presentation
- S5. Average go responses per subject
- S6, S7. Scratching during JBT
- S8. Scent-marking during JBT
-
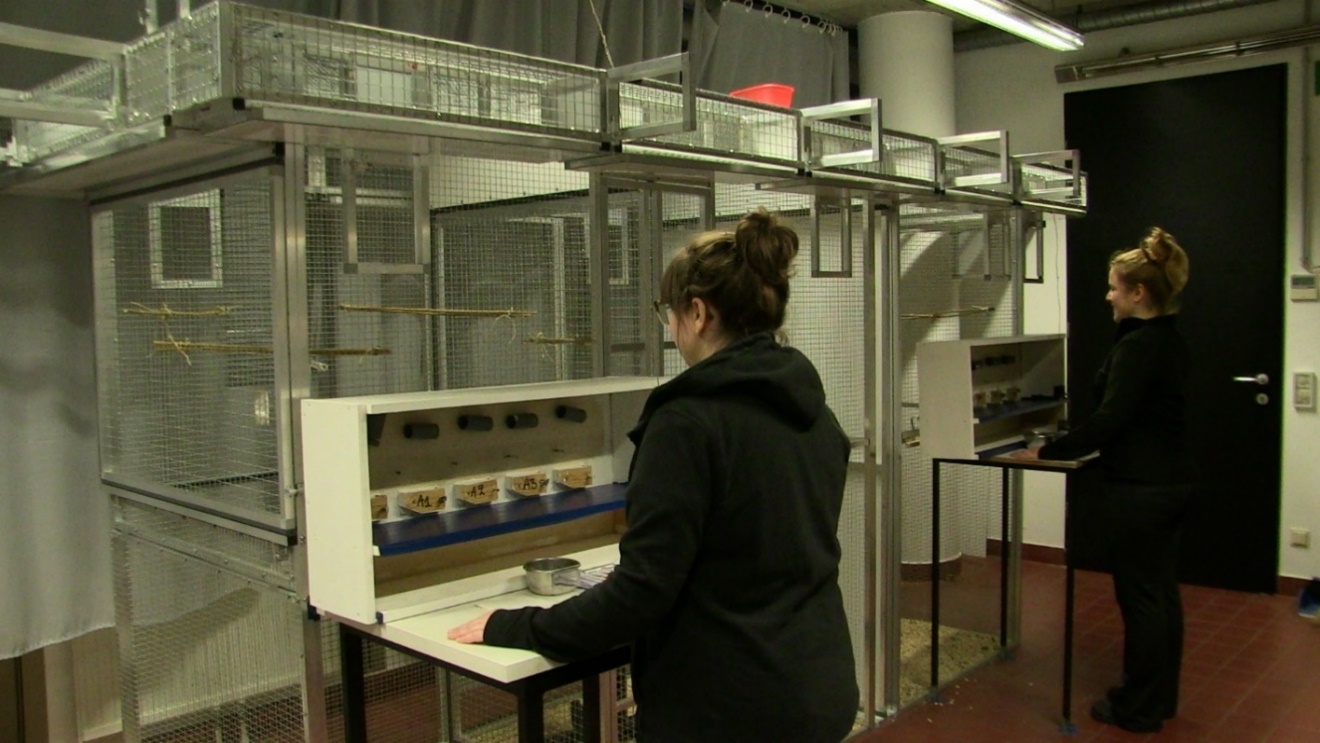
S9. Gnawing during JBT

**Figure S1. Experimental design.** From left to right: demonstrator’s compartment, stimulus presentation compartment (with red container on top, filled with food for odour control), white moveable door, and observer’s compartment. Both demonstrator’s and observer’s compartment had a JBT apparatus placed in front (size apparatus: 84 x 20 x 40 cm). This design allowed for two researchers to conduct the JBT at the same time with the two participating subjects. Both compartments were designed identically (size: 100 x 100 x 200 cm; distance to trial initiator: 100 cm). The tunnels with moveable doors at the top of the experimental cage provided access to the compartments. The door between the middle and observer’s compartment remained closed during the JBT’s, and was only opened during the emotion manipulation.


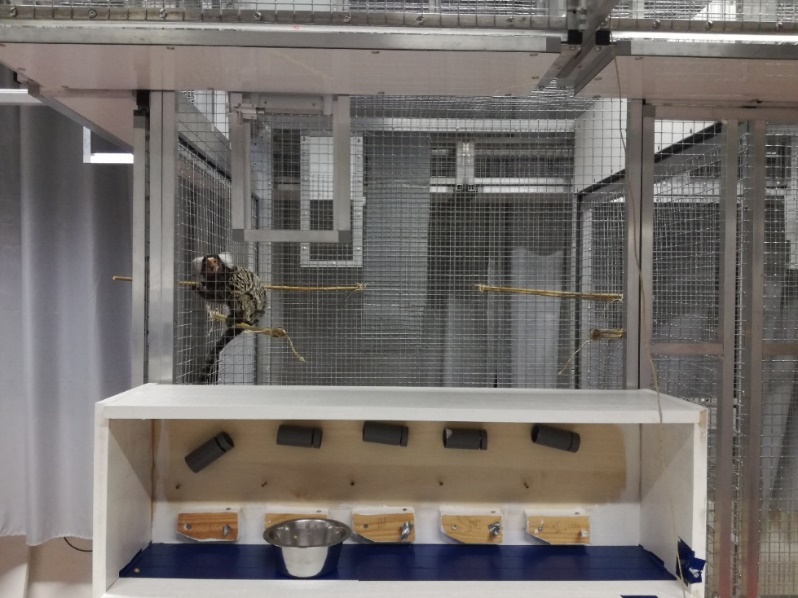


**Figure S2. Front view of demonstrators’ compartment.** The judgement bias apparatus with 5 door cues representing the positive P cue, near-positive NP cue, middle M cue, near-negative NN cue, and the negative N cue. A string, located on the right side of the apparatus, connected the apparatus and the trial initiator (on the other side).


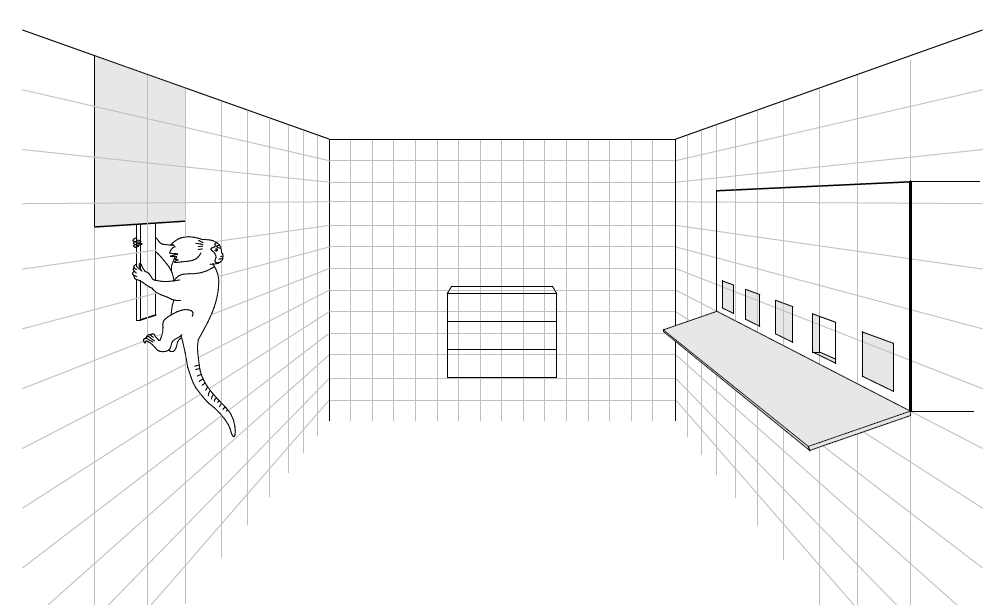


**Figure S3. Side view of demonstrator’s compartment**. The subject is touching the trial initiator, which can then be pulled up by a string, after which the initiator disappears behind a (grey) view blocker. On the right is the judgement bias test apparatus. To go from the initiator to the test, subjects had the option to jump across or use the left or right side of the wire mesh cage. In front of the test apparatus a platform was attached on which subjects could land and move during the test.


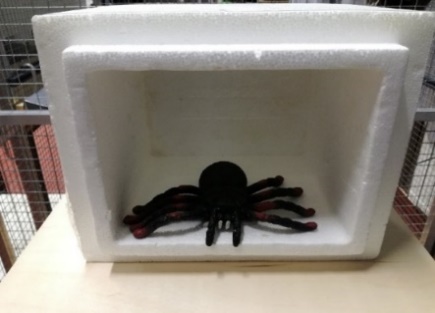

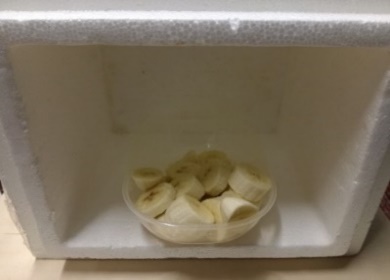

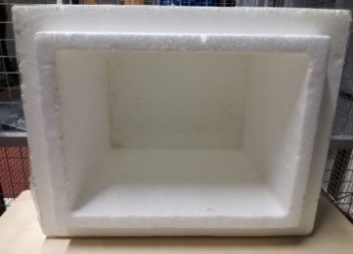


**Figure S4. Stimulus presentation.** Left: negative condition, large artificial rubber spider. Middle: control condition, empty box. Right: positive condition, preferred food cut into pieces.


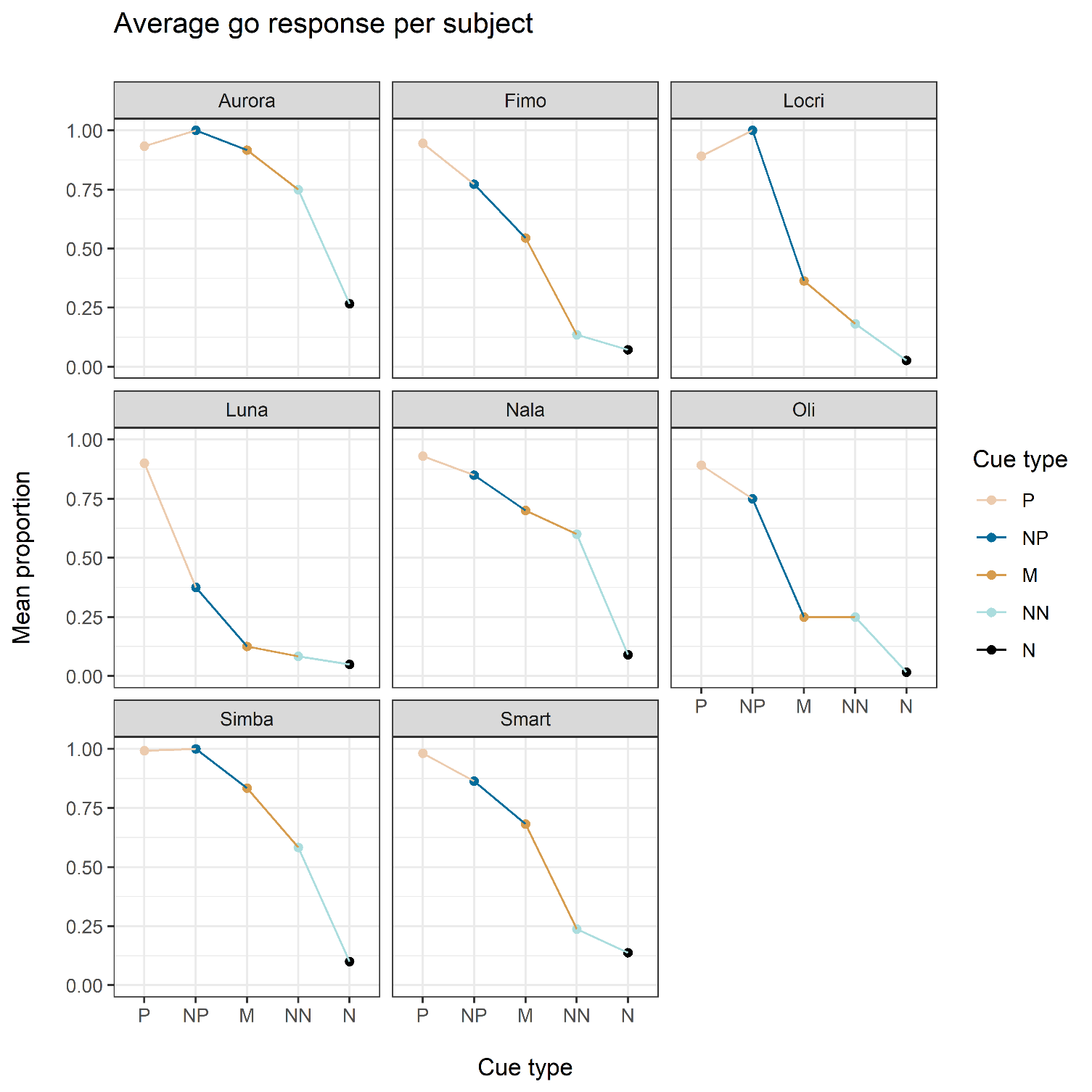


**Figure S5. Average raw proportion of go responses per subject showing a monotonic graded curve.** P= positive cue; NP= near positive cue; M= middle cue; NN= near negative cue; N= negative cue


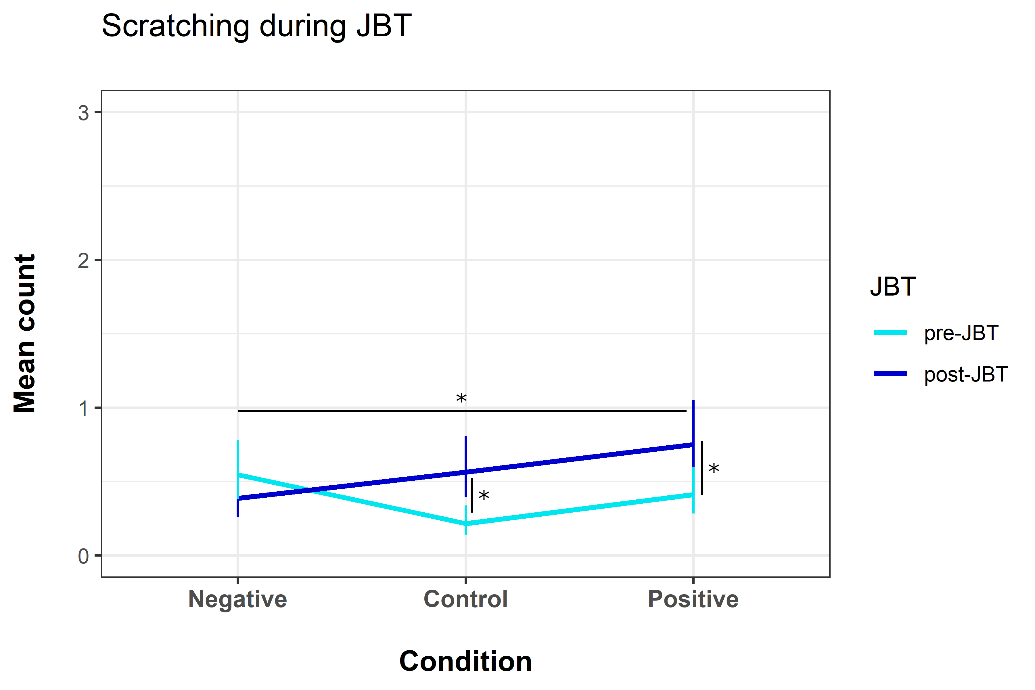


**Figure S6. Mean predicted count scratching** (bars indicate SE). * P < 0.05


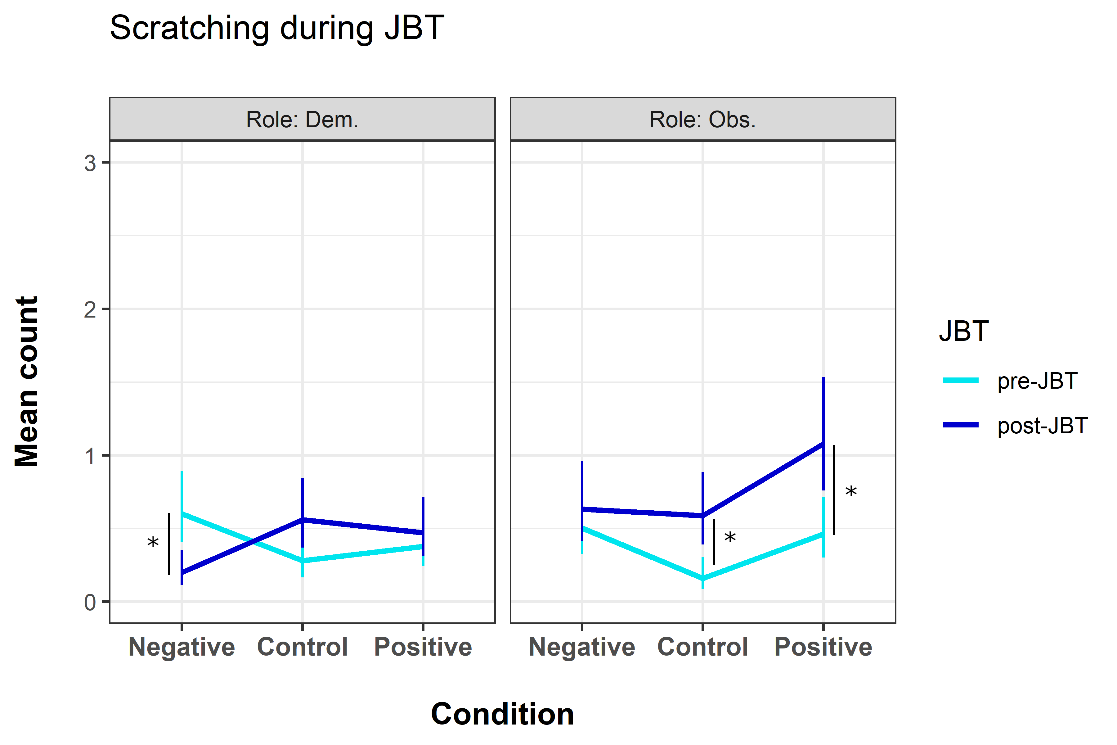


**Figure S7. Mean predicted count scratching** (bars indicate SE): between roles (demonstrator, observer) and across conditions (negative, control, positive), for each judgement bias test (pre-JBT and post-JBT). * P < 0.05


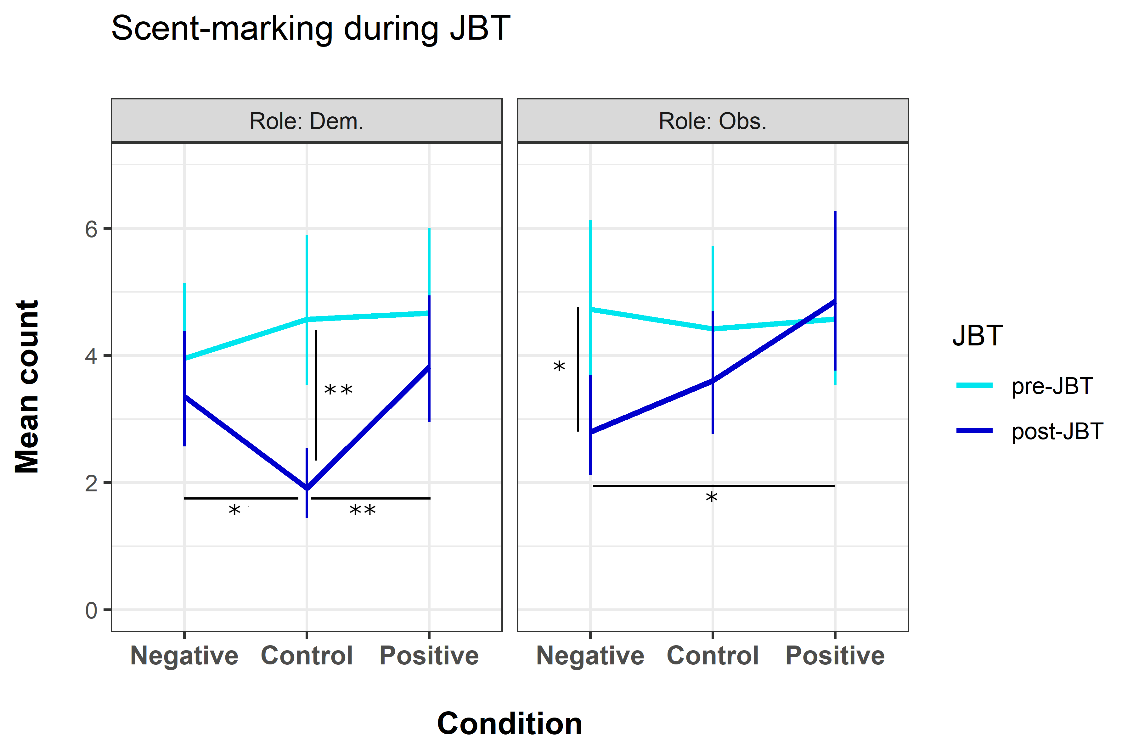


**Figure S8. Mean predicted count scent marking** (bars indicate SE): between roles (demonstrator, observer) and across conditions (negative, control, positive), for each judgement bias test (pre-JBT and post-JBT). * P < 0.01, ** P < 0.001


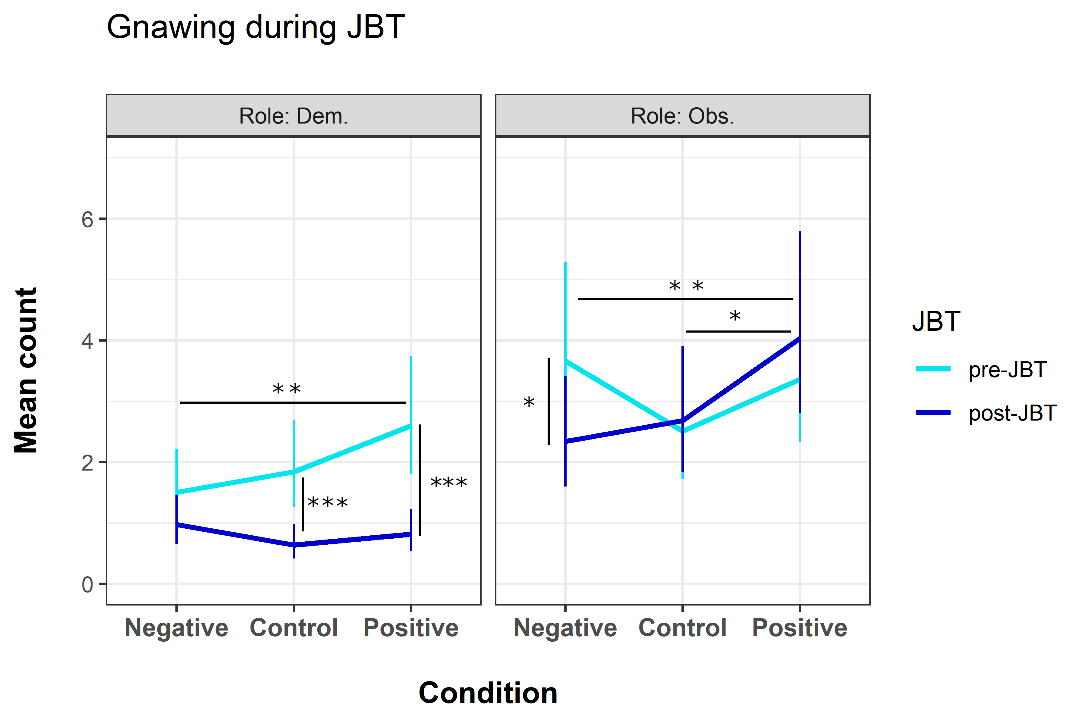


**Figure S9. Mean predicted count gnawing** (bars indicate SE): between roles (demonstrator, observer) and across conditions (negative, control, positive), for each judgement bias test (pre-JBT and post-JBT). *** P < 0.001, ** P < 0.01, * P < 0.05
