# Supplementary material for: Watching others in a positive state does not induce optimism bias in common marmosets (Callithrix jacchus), but leads to behaviour indicative of competition
Source: Anim Cogn. 2021 Mar 16;24(5):1039–56. doi: 10.1007/s10071-021-01497-1 (PMC8360889; doi:10.1007/s10071-021-01497-1)
Supplement: Supplementary file 4 — Supplementary file4 (DOCX 22 KB) [file 10071_2021_1497_MOESM4_ESM.docx]

**Supplementary Tables -** *Watching others in a positive state does not induce optimism bias in common marmosets (Callithrix jacchus), but leads to behaviour indicative of competition.* Adriaense J.E.C., Šlipogor V., Hintze S., Marshall L., Lamm C., Bugnyar T.

- S1. Subject details
- S2. Training schedule
- S3. Ethogram
- S4. Total test trials per subject
- S5. Chronological order of testing per subject
- S6. Training results per subject

**Table S1. Subject details**

| **Subject** | **Sex** | **Age at time of study** | **Social group*** | **Relatedness** |
| --- | --- | --- | --- | --- |
| Aurora | F | 5 | 1 | NA (i.e. single testing) |
| Oli | F | 12 | 2 | Mother |
| Luna | F | 4 | 2 | Daughter |
| Locri | M | 14 | 3 | Siblings |
| Fimo | M | 15 | 3 |  |
| Smart | M | 8 | 4 | Father |
| Simba | M | 2 | 4 | Son of Smart + sibling of Nala |
| Nala | F | 2 | 4 | Daughter of Smart + sibling of Simba |

*Social group refers to the group the subject is housed in, and with which other study subjects they share their housing

**Table S2. Training schedule**

| **Training steps** | | | | **Criterion** |
| --- | --- | --- | --- | --- |
| **1. Apparatus training** | **1.Initiator training** | **1.Trial initiator**  **(by hand)** | 1. Reward placed on top of trial initiator | Open end |
|  |  |  | 2. Reward given after touching trial initiator | Open end |
|  |  |  | 3. Reward given at increasing distance (from 10 cm to 100 cm) | Open end |
|  |  | **2. Trial initiator**  **(by string)** | 1. Reward given after touching trial initiator and coming to the opposite side of the cage | 20 trials |
|  | **2. Door training** | **1. Shaping P door** | 1. Reward given after touching trial initiator and coming to the P* door. | Min. 20 trials |
|  |  | **2. Shaping P and N doors** | 1. Reward given after touching trial initiator and coming to either the P or N door | 15 P, 15 N; 80% correct Go; within 10 s. |
| **2. Discrimination training** | | Reward given after touching trial initiator and coming to the P door. No reward was given when coming to the N door. | | 10 P, 10 N; 80% correct Go per cue/per day; within 10 s; 3 consecutive days |

^*^P= positive cue or door; N= negative cue or door

**Table S3. Ethogram of behavioural variables.**

| **Behaviour** | **Description** |
| --- | --- |
| Go response | Subject sits directly in front of the open apparatus door within 10 seconds after touching the trial initiator. |
| No-go response | Subject does not sit in front of the open apparatus door within 10 seconds after touching the trial initiator, or they sit in front of a closed (i.e. wrong) door. |
| Active choice | Subject touches the trial initiator again, within 10 seconds after touching it previously, without first going to the open door. This response also codes as “no-go response”. |
| Wrong choice | Subject either performs a go response in an N trial or a no-go response in a P trial. |
| Scent marking | Marmoset rubs sternal or anogenital area over surface. |
| Gnawing | Marmoset gnaws at wood with teeth (i.e. also known as “gouging”). |
| Scratching | Marmoset repeatedly moves hand or foot with claws drawn rapidly across fur. |
| Pilo-erected tail | Brushed or raised fur on tail. |
| Position | The position refers to the subject either staying directly in front of the presented stimulus, or away from it |
| Positive calls | Combination of “chirp” and food-beg calls |
| Negative calls | Combination of “tsik”, “tsik-egg”, “cough”, and “seep” calls |
| Egg call | Also called eck or ek calls in literature |
| Contact call | Combination of “phee”, “shrill”, and “whirr” calls |

**Table S4. Total test trials per subject**

| **Subject** | **Test period^1^** | **Total trials P^2^** | **Total trials NP** | **Total trials M** | **Total trials NN** | **Total trials N** | **Total trials^3^** |
| --- | --- | --- | --- | --- | --- | --- | --- |
| **Aurora** | **1** | 30 | 6 | 6 | 6 | 30 | 78 |
|  | **2** | 30 | 6 | 6 | 6 | 30 | 78 |
| **Oli** | **1** | 60 | 12 | 12 | 12 | 60 | 156 |
|  | **2** | 60 | 12 | 12 | 12 | 60 | 156 |
| **Luna** | **1** | 60 | 12 | 12 | 12 | 60 | 156 |
|  | **2** | 60 | 12 | 12 | 12 | 60 | 156 |
| **Fimo** | **1** | 60 | 12 | 12 | 12 | 60 | 156 |
|  | **2** | 50 | 10 | 10 | 10 | 50 | 130 |
| **Locri** | **1** | 60 | 12 | 12 | 12 | 60 | 156 |
|  | **2** | 50 | 10 | 10 | 10 | 50 | 130 |
| **Smart** | **1** | 58 | 12 | 12 | 11 | 59 | 152 |
|  | **2** | 50 | 10 | 10 | 10 | 50 | 130 |
| **Simba** | **1** | 60 | 12 | 12 | 12 | 59 | 155 |
|  | **2** | 60 | 12 | 12 | 12 | 60 | 156 |
| **Nala** | **1** | 50 | 10 | 10 | 10 | 50 | 130 |
|  | **2** | 50 | 10 | 10 | 10 | 50 | 130 |

^1^Test period= refers to testing in time period 1 or 2, in which period 2 is repeated measures testing of period 1 (see also info under Methods in main paper).

^2^P= positive cue; NP= near positive cue; M= middle cue; NN= near negative cue; N= negative cue

^3^Number of test trials differs between animals as not all subjects finished testing within the designated timeframe of our study

**Table S5. Chronological order of testing per subject**

|  |  | **Demonstrator** | | | **Observer** | | |
| --- | --- | --- | --- | --- | --- | --- | --- |
| **Subject** | **Test period** | **Positive condition** | **Negative condition** | **Control condition** | **Positive condition** | **Negative condition** | **Control condition** |
| **Aurora** | **1** | 1 | 5 | 2 | *NA* | | |
|  | **2** | 3 | 6 | 4 |  |  |  |
| **Oli** | **1** | 3 | 1 | 5 | 4 | 2 | 6 |
|  | **2** | 7 | 12 | 10 | 9 | 8 | 11 |
| **Luna** | **1** | 4 | 2 | 6 | 3 | 1 | 5 |
|  | **2** | 9 | 8 | 11 | 7 | 12 | 10 |
| **Fimo** | **1** | 4 | 3 | 1 | 2 | 10 | 5 |
|  | **2** | 8 | 9 | 6 | 11 | *NA* | 7 |
| **Locri** | **1** | 2 | 10 | 5 | 4 | 3 | 1 |
|  | **2** | 11 | *NA* | 7 | 8 | 9 | 6 |
| **Smart** | **1** | 3 | 1 | 4 | 2 | 8 | 5 |
|  | **2** | 10 | 6 | *NA* | 7 | 11 | 9 |
| **Simba** | **1** | 3 | 6 | 1 | 4 | 5 | 2 |
|  | **2** | 12 | 11 | 9 | 7 | 10 | 8 |
| **Nala** | **1** | 3 | 8 | 1 | 2 | 7 | 6 |
|  | **2** | 4 | 10 | 5 | 9 | *NA* | *NA* |

**Table S6. Training data per subject** (see Table S2 for description of training steps)

| **Subject** | **1. Initiator training** *(total trials)* | | **2. Door training**  *(total trials)* | | **3. Discrimination training** | | |
| --- | --- | --- | --- | --- | --- | --- | --- |
|  | **1.Initiator by hand** | **2. Initiator by string** | **1. Shaping P door** | **2. Shaping P and N doors** | **Number of trained days before reaching criterion** | **Number of trained trials** | **% correct response per cue (***results of correct 3 consecutive days shown***)** |
| **Aurora** | 99 | 108 | 24 | 90 | 5 | 100 | 100% P, 93% N |
| **Oli** | 77 | 154 | 30 | 58 | 9 | 180 | 93% P; 90% N |
| **Luna** | 103 | 130 | 25 | 86 | 5 | 100 | 95% P; 90% N |
| **Fimo** | 144 | 85 | 29 | 50 | 8 | 160 | 100% P; 100% N |
| **Locri** | 179 | 124 | 20 | 92 | 5 | 100 | 90% P; 95% N |
| **Smart** | 150 | 130 | 27 | 60 | 8 | 160 | 95% P; 95% N |
| **Simba** | 157 | 91 | 20 | 74 | 8 | 160 | 100% P; 93% N |
| **Nala** | 150 | 68 | 34 | 62 | 3 | 60 | 95% P; 90% N |
